# NIR‐Light Activable 3D Printed Platform Nanoarchitectured with Electrospun Plasmonic Filaments for On Demand Treatment of Infected Wounds

**DOI:** 10.1002/adhm.202404274

**Published:** 2024-12-25

**Authors:** Daniel Rybak, Jingtao Du, Paweł Nakielski, Chiara Rinoldi, Alicja Kosik‐Kozioł, Anna Zakrzewska, Haoyang Wu, Jing Li, Xiaoran Li, Yunlong Yu, Bin Ding, Filippo Pierini

**Affiliations:** ^1^ Department of Biosystems and Soft Matter Institute of Fundamental Technological Research Polish Academy of Sciences Warsaw 02‐106 Poland; ^2^ Innovation Center for Textile Science and Technology College of Textiles Donghua University Shanghai 201620 P. R. China; ^3^ Institute of Burn Research Southwest Hospital Third Military Medical University (Army Medical University) Chongqing 400038 P. R. China

**Keywords:** 3D printing, infected wound healing, NIR light‐responsive, plasmonic short‐filaments, smart drug delivery

## Abstract

Bacterial infections can lead to severe complications that adversely affect wound healing. Thus, the development of effective wound dressings has become a major focus in the biomedical field, as current solutions remain insufficient for treating complex, particularly chronic wounds. Designing an optimal environment for healing and tissue regeneration is essential. This study aims to optimize a multi‐functional 3D printed hydrogel for infected wounds. A dexamethasone (DMX)‐loaded electrospun mat, incorporated with gold nanorods (AuNRs), is structured into short filaments (SFs). The SFs are 3D printed into gelatine methacrylate (GelMA) and sodium alginate (SA) scaffold. The photo‐responsive AuNRs within SFs significantly enhanced DXM release when exposed to near‐infrared (NIR) light. The material exhibits excellent photothermal properties, biocompatibility, and antibacterial activity under NIR irradiation, effectively eliminating *Staphylococcus aureus* and *Escherichia coli in vitro*. In vivo, material combined with NIR light treatment facilitate infectes wound healing, killing *S. aureus* bacteria, reduced inflammation, and induced vascularization. The final materials’ shape can be adjusted to the skin defect, release the anti‐inflammatory DXM on‐demand, provide antimicrobial protection, and accelerate the healing of chronic wounds.

## Graphical abstract

This study shows the development of multi‐functional 3D printed hydrogel composite for infected wound healing. Drug‐ and AuNRs‐loaded polymeric short‐filaments can, on demand, deliver the anti‐inflammatory drug and eradicate the bacteria. In vivo shows that material promotes bacterial eradication, reduces inflammation, and induces vascularization, accelerating wound healing and highlighting its therapeutic potential.

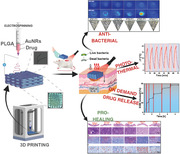

## 1 Introduction

Wound healing presents a significant clinical challenge in contemporary medicine due to its complexity.^[^ ^1^ ^]^ It involves dynamic and spatiotemporal phases that require various multifaceted strategies activated at a specified time.^[^ ^2^ ^]^ However, when affected by pathogenic factors while its integrity is disrupted, it makes the epidermis susceptible to inflammation and infection progression.^[^ ^3^ ^]^ This hinders the advancement of the wound repair process and increases the risk of subsequent septicemia.^[^ ^4^ ^]^

Chronic wounds are an urgent problem that must be addressed. While current dressings have enhanced wound healing outcomes, they remain inadequate for chronic ones.^[^ ^5^ ^]^ As a result, there is an urgent need to develop novel materials to improve the treatment of chronic wounds. They usually disrupt the inflammatory process by extending its duration and reducing responsiveness, preventing the transition to the proliferation phase. Prolonged inflammation in the wound microenvironment can be caused by varied factors, such as bacterial infection.^[^ ^6^, ^7^ ^]^ Typical antibacterial materials include the employment of antibiotics, metal ions (e.g., Ag^+^, Au^+^), or antibacterial peptides.^[^ ^8^ ^]^ However, the recent increase in bacterial resistance to antibacterial agents represents a significant menace to human health.^[^ ^9^ ^]^ Therefore, novel antibacterial strategies are required.^[^ ^3^ ^]^ Thus, laser‐assisted photothermal therapy (PTT) has emerged to face bacterial infection challenges.^[^ ^10^ ^]^ PTT converts the near‐infrared (NIR) light into heat via photo‐thermal agents such as gold nanorods (AuNRs). AuNRs absorb the light in the NIR window (700 to 950 nm) due to the localized surface plasmon resonance phenomenon, which depends mainly on AuNR size and shape.^[^ ^11^ ^]^ The rapid rise in local temperature after laser exposure can effectively kill the bacteria.^[^ ^12^, ^13^ ^]^

Electrospinning is one of the most promising techniques used for the preparation of regenerative medicine and wound healing materials. Electrospun polymers’ morphology mimics the properties of the extracellular matrix, which is essential for enhancing the efficacy of diverse therapeutic approaches. Moreover, their high aspect ratio between big surface area and small diameter makes them ideal for drug delivery.^[^ ^14^ ^]^

Incorporating AuNRs into the electrospun polymer allows the production of stimuli‐responsive material, to deliver the drug on‐demand.^[^ ^15^ ^]^ Adding anti‐inflammatory DXM can create an on‐demand drug delivery system combining antibacterial properties.^[^ ^16^, ^17^ ^]^ An optimal material for chronic wound healing should offer adjustable and controllable release kinetics for the drug to ensure effective and safe therapeutic effects.^[^ ^18^, ^19^, ^20^, ^21^ ^]^ AuNRs can accelerate the release from the carrier by generating local heat through NIR laser irradiation.^[^ ^22^ ^]^ Although polymeric materials can deliver lipophilic drugs, their water affinity is poor. Therefore, hydrogels gained interest in wound healing applications due to their capability to absorb substantial amounts of water and biological fluids.^[^ ^23^ ^]^ They can protect the drug from the surroundings and have adjustable properties, coupled with the ability to retain a significant fraction of solvents.^[^ ^24^ ^]^ Despite modifying hydrogel properties that can control the release, their low affinity to most drugs and big pores lead to a high percentage of release ratio within a short time.^[^ ^25^ ^]^

Thus, numerous studies have been reported discussing the development of hydrogels with embedded fibrous membranes.^[^ ^26^, ^27^, ^28^, ^29^ ^]^ However, the biggest challenge is their structural integrity. Due to the limited interaction between the fibrous mat and the hydrogel matrix, it often results in delamination. Nevertheless, significant progress has been made in the short filaments (SFs) incorporated into hydrogel networks to address these limitations.^[30‐33]^ Broda *et al.* introduced a novel method for preparing polycaprolactone (PCL) SFs using cryo‐cutting of aligned polymer fibrous mats. The cryocut SFs demonstrated excellent dispersion without any agglomeration.^[^ ^34^ ^]^ The low‐temperature conditions and short preparation time have a promising potential for drug‐containing SFs preparation.^[^ ^35^, ^36^ ^]^

A promising technique that may combine stimuli‐responsive small‐size SFs within a hydrogel network is 3D printing.^[^ ^37^ ^]^ Considering the personalized demand, 3D printing can be applied to tailor the hydrogels into highly organized structures to match the size and depth of a specific wound.^[^ ^38^ ^]^ These 3D‐printed scaffolds possess a porous structure with interconnected pores, promoting cell migration and growth, the transport of nutrients, oxygen, and metabolic waste, and aiding in the removal of wound exudates. Advanced composite materials with a well‐organized, heterogeneous porous architecture can mimic the complex physiological and morphological structures of biological tissues, enhancing wound healing.^[^ ^39^ ^]^

**Scheme 1:**
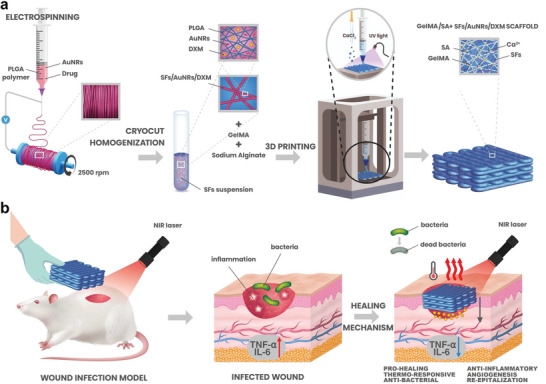
Illustration representing a) preparation process of the material and its composition and b) main application and mechanism of wound healing.

This study reports the development of stimuli‐responsive 3D printed scaffolds that feature on‐demand drug delivery and antibacterial properties. We presented a complex GelMA and SA material, which holds potential for use in treating infected wounds (**Scheme** **1**a). Adding SFs containing AuNRs and DXM slightly improved the mechanical properties. Additionally, the hydrogel increased the local temperature generated by near‐infrared (NIR) light, eliminating *S. aureus* and *E. coli*. Repetitive NIR exposure further promoted DXM release by raising the temperature, leading to controlled and gradual release of the drug. We also proposed a mechanical approach for producing stimuli‐responsive SFs containing active agents while minimizing the loss of the drug. The electrospinning technique prepared an aligned poly(D, L‐lactide‐*co*‐glycolide) (PLGA) mat containing AuNRs and DXM. The mat was cut using cryo‐cutting and homogenized, followed by washing and lyophilization. The SFs powder was mixed with GelMA/SA and 3D printed using the extrusion printing method combined with UV curing and CaCl_2_ to start the hydrogel cross‐linking. Furthermore, hydrogels proved to be biocompatible and showed good cell infiltration. In vivo studies showed that combining the hydrogel with NIR light promotes the healing of infected wounds. The final materials’ shape can be adjusted to the skin defect, release the anti‐inflammatory DXM on‐demand, provide antimicrobial protection, and accelerate the healing of chronic wounds (Scheme 1b).

## 2 Results and Discussion

In this study, we developed a photo‐responsive 3D printed scaffold by combining GelMA and SA, resulting in a stable construct. Hydrogels were incorporated with electrospun photo‐responsive and drug‐containing polymeric SFs. AuNRs within the fiber structure increased the temperature through NIR light‐heat conversion. Additionally, repeated NIR treatments accelerated the release of DXM, leading to a gradual drug release pattern. Photo‐thermal heat generated after NIR laser exposure eradicated *S. aureus* and *E. coli* bacteria in >99.9% and 99.9%, respectively. Moreover, In vivo experiments demonstrated that the hydrogel composites combined with NIR light treatment facilitated infected wound healing, killing *S. aureus* bacteria, reducing inflammation, and inducing vascularization of wound beds.

### 2.1 Short Filaments Characterization

**Figure 1:**
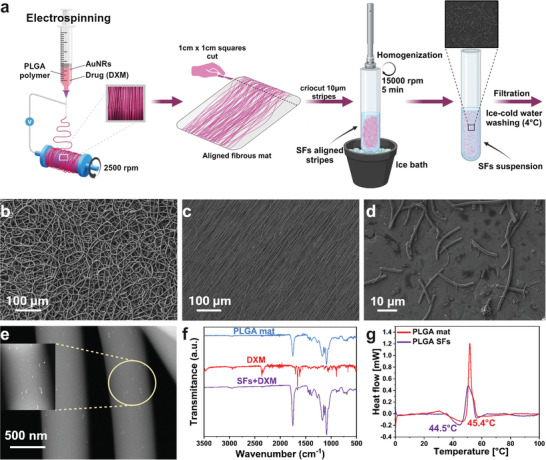
Electrospun fibers characterization. a) Schematic representation of the SFs preparation process. b) FE‐SEM of random fiber PLGA mat. c) FE‐SEM visualizing the aligned PLGA mat. d) FE‐SEM visualization of single fibers morphology after the structurization process. e) TEM image of AuNRs dispersed inside the fiber structure. f) FTIR analysis of DXM‐incorporated SFs showing the drug presence after material structurization. g) DSC analysis of the polymeric material indicating the Tg before (45.5°C) and after (44.3 °C) SFs preparation.

According to our previous work, we prepared the DXM and AuNRs containing SFs using the cryocut technique (**Figure** **1**a).^[^ ^40^ ^]^ PLGA was selected as a polymeric material due to its advantageous characteristics, including biocompatibility, biodegradability, and the possibility of controlling the drug release from the matrix.^[^ ^41^, ^42^ ^]^ PLGA fibers free from beads and branching were prepared by electrospinning (Figure 1b). Previous research has demonstrated that fiber alignment and the absence of branching contribute to the effective dispersion of fibers into individual filaments.^[^ ^40^, ^43^ ^]^ A highly aligned polymeric mat was produced (Figure 1c). The cryocut process, followed by washing and lyophilization, was applied for SFs preparation. The single SFs showed decent uniformity and no agglomeration, and their structure remained unchanged due to mechanical structurization (Figure 1d). The TEM imaging was utilized to confirm the presence of AuNRs within the fiber structure after the electrospinning process. Figure 1e shows the AuNRs eventually dispersed within the spun fibers. The FTIR analysis for PLGA material before cutting, PLGA SFs containing DXM, and DXM powder were also performed (Figure 1f). The PLGA mats’ spectrum displayed characteristic peaks at 2999 cm^−1^ and 2954 cm^−1^, representing stretching vibrations of the ‐CH, ‐CH_2_, and ‐CH_3_ functional groups. Additionally, a peak ≈1729 cm^−1^ indicated C═O stretching. The bond at 1093 cm^−1^ was linked to the symmetrical stretching of the ─C═O, while the 1172 cm^−1^ peak was associated with the asymmetrical stretching of the C─O─C bond.^[^ ^41^, ^43^ ^]^ The peaks at 1696, 1671, and 1591 cm^−1^ are associated with the stretching vibration of ─C═O, linked to the C_3_‐cyclic and C_20_ carbonyl group, as well as the conjugated double bond framework with ─C═O bonds.^[44]^ A notable absorption band at 3476 cm^−1^ was observed, corresponding to the O‐H bond stretching vibration.^[^ ^45^, ^46^ ^]^ To verify that the structurization of PLGA into SFs does not alter the DXM, the spectra of SFs+DXM were analyzed. The 1671 and 1591 cm^−1^ characteristic stretching vibration of ─C ═O DXM bands remained unaffected due to the processing and remained as a combination of individual components. PLGA polymer and DXM structures showed no variations before and after structurization. To understand how temperature influences drug release from the polymer, the DSC analysis was performed, probing the polymers’ Tg. Tg is a temperature threshold at which polymer transitions from rigid glass into a rubbery state.^[^ ^47^, ^48^ ^]^ Studies have shown that maintaining a temperature below a certain Tg ensures sustained release, avoiding an undesirable initial burst release.^[^ ^49^, ^50^ ^]^ A slight difference in Tg was observed between the electrospun PLGA mat (45.5 °C) and SFs (44.3 °C) (Figure 1g). The measured distinction in Tg is critical for controlling the release of DXM from the SFs and the final photo‐responsive hydrogel, as exceeding 44.3 °C will significantly impact the release profile.

### 2.2 3D Scaffold Characterization

**Figure 2:**
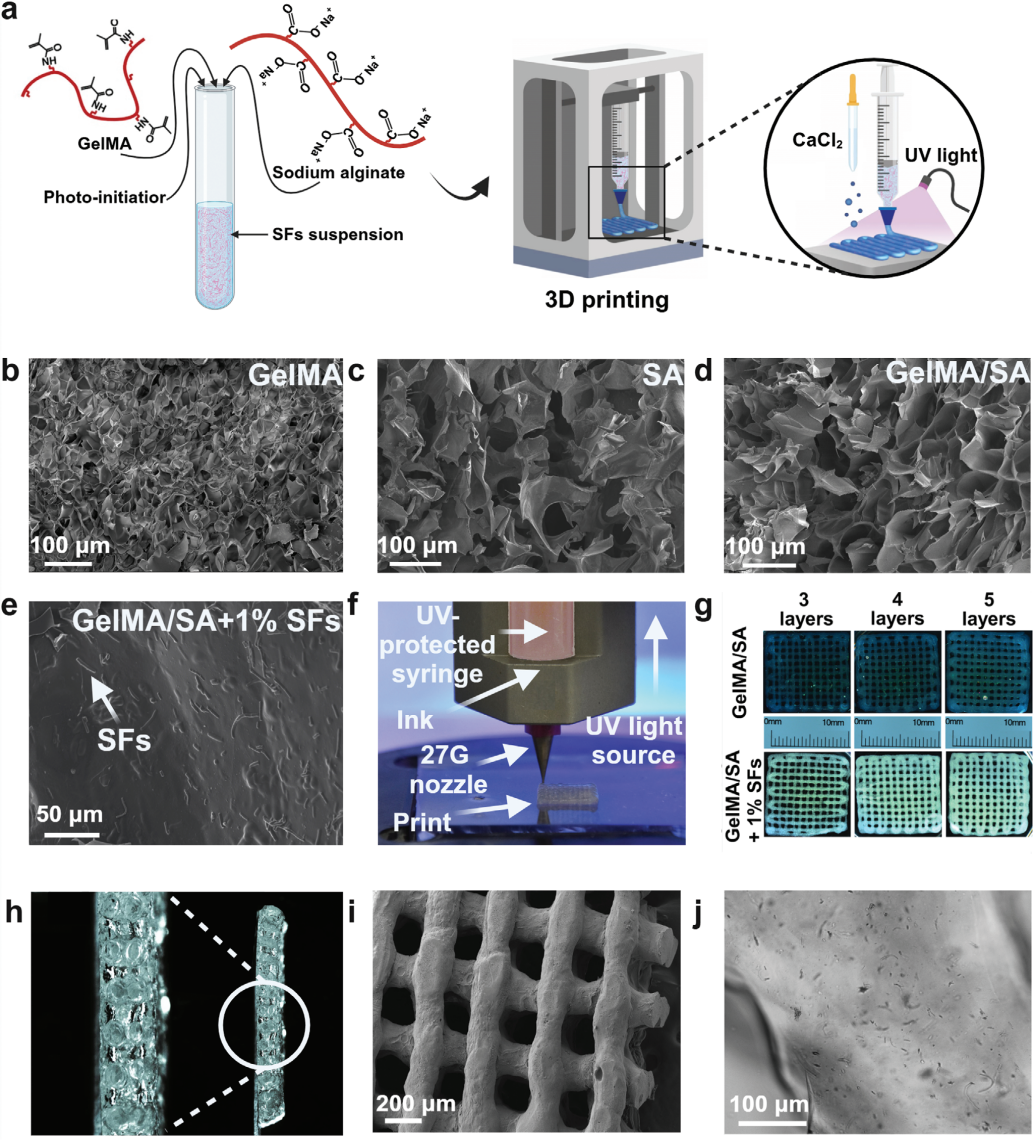
Characterization of the 3D printed hydrogel composite. a) Scheme representing 3D printed material preparation b) SEM image of GelMA component. c) SEM image of the SA component. d) SEM image of the GelMA/SA composite. e) SEM representing the cross‐linked GelMA/SA component with evenly dispersed 1% SFs (w/w) within the hydrogel matrix. f) Photo representing the 3D printer machine used for the scaffold preparation. g) Stereoscope photo panel showing the structure of a different material composition – layers and SFs influence material thickness. h) Cross‐section of 5 layered prints showing round filament structure. i) FE‐SEM image of the printed structure with a PI ≈ 1.0021. j) 3D printed single filament revealed the presence of SFs within its structure.

The SEM was used to evaluate the effect of each component on the GelMA/SA hydrogel network. Prepared hydrogels possess a microstructure with micron‐sized pores. This feature is essential for maintaining a significant water volume and improving the capacity to load and disperse SFs.^[^ ^51^ ^]^ SEM analysis revealed a spongy‐like surface containing a small number of macropores and vast amount of micropores alongside the GelMA structure (**Figure** **2**b). The SEM images of SA showed a porous structure with a visibly more significant macroporous composition (Figure 2c). The composite hydrogel revealed the presence of well‐defined, interconnected voids and spacious pores within the network, corroborating the interchain interactions between the hydrogel's building blocks (Figure 2d). Hydrogels necessitate an interconnected network of pores as this architecture serves as the groundwork for efficiently transporting oxygen, nutrients, and metabolites.^[^ ^52^ ^]^ The internal microstructure with micron‐sized pores replicates the extracellular matrix (ECM) and is critical in facilitating biological processes and promoting tissue regeneration.^[^ ^53^ ^]^ To investigate the impact of individual constituents on the overall porosity of the material, the average pore diameter within each component and the final composite was quantified (Figure S1, Supporting Information). The average pore size is 23.8 ± 6.3 µm, 80.1 ± 26.5 µm, and 57.4 ± 15.2 µm for GelMA, SA, and GelMA/SA, respectively. The literature reports highlighting the impact of implant pore size on tissue regeneration, showing that an optimum pore size of 20–125 µm is ideal for the regeneration of adult mammalian skin. The SA introduction into GelMA increased hydrogel pore size significantly (*p* ≤ 0.0001; n = 20), highlighting SA's crucial influence on the final material's morphological characteristics that can possibly support the regeneration of skin tissue.^[^ ^55^, ^56^ ^]^

Moreover, the GelMA/SA incorporated with 1% of SFs was visualized under the SEM to evaluate the dispersion of the fibers within the hydrogel network (Figure 2e). The image revealed well‐dispersed single fibers with no significant visible agglomerations.

Bioinks used in 3D printing are widely recognized as critically important for fabricating 3D scaffolds for tissue regeneration. 3D printing technology offers a transformative platform for developing personalized and bespoke therapeutic approaches equipped, for example, with optimized drug delivery systems, resulting in a revolutionary advance in healthcare.^[^ ^57^ ^]^ Thus, we printed hydrogel materials using a simple extrusion‐based printer (Figure 2f). The prints were UV‐ and CaCl_2_‐cured during the process. Different layers and sizes of 1 cm x 1 cm scaffolds were printed to optimize the process (Figure 2g). The average filament width of the printed hydrogel scaffolds was between 380.3 ± 43.5, 411.1 ± 35.5, and 454.6 ± 34.3 µm for 3 layers, 4 layers, and 5 layers without SFs, respectively (Figure S2, Supporting Information). After SFs incorporation, the average filament width was 370.3 ± 41.4, 474.0 ± 41.5, and 493.1 ± 45.0 µm for 3 layers, 4 layers, and 5 layers, respectively. No statistical differences were found after incorporating SFs into 3 layered prints. However, slight differences can be seen in 4‐layered (*p* ≤ 0.01) and 5‐layered (*p* ≤ 0.05) prints. The cross‐section of the scaffold showed a well‐defined, porous, and round structure of single‐printed filaments (Figure 2h). The SEM images of dehydrated and fixed structures confirmed the round shape of print filament with squared holes formed between them, indicating printability index (PI) close to 1 (Figure 2i). When the ideal gelation conditions appear, the internal printed channel of the scaffold is square, and the PI takes a value equal to 1, as mentioned before. The optical stereoscope was employed to visualize the SFs inside the printed filaments (Figure 2j). It shows that the polymer fibers are homogenously dispersed within the hydrogel network.

### 2.3 Mechanical Tests

**Figure 3:**
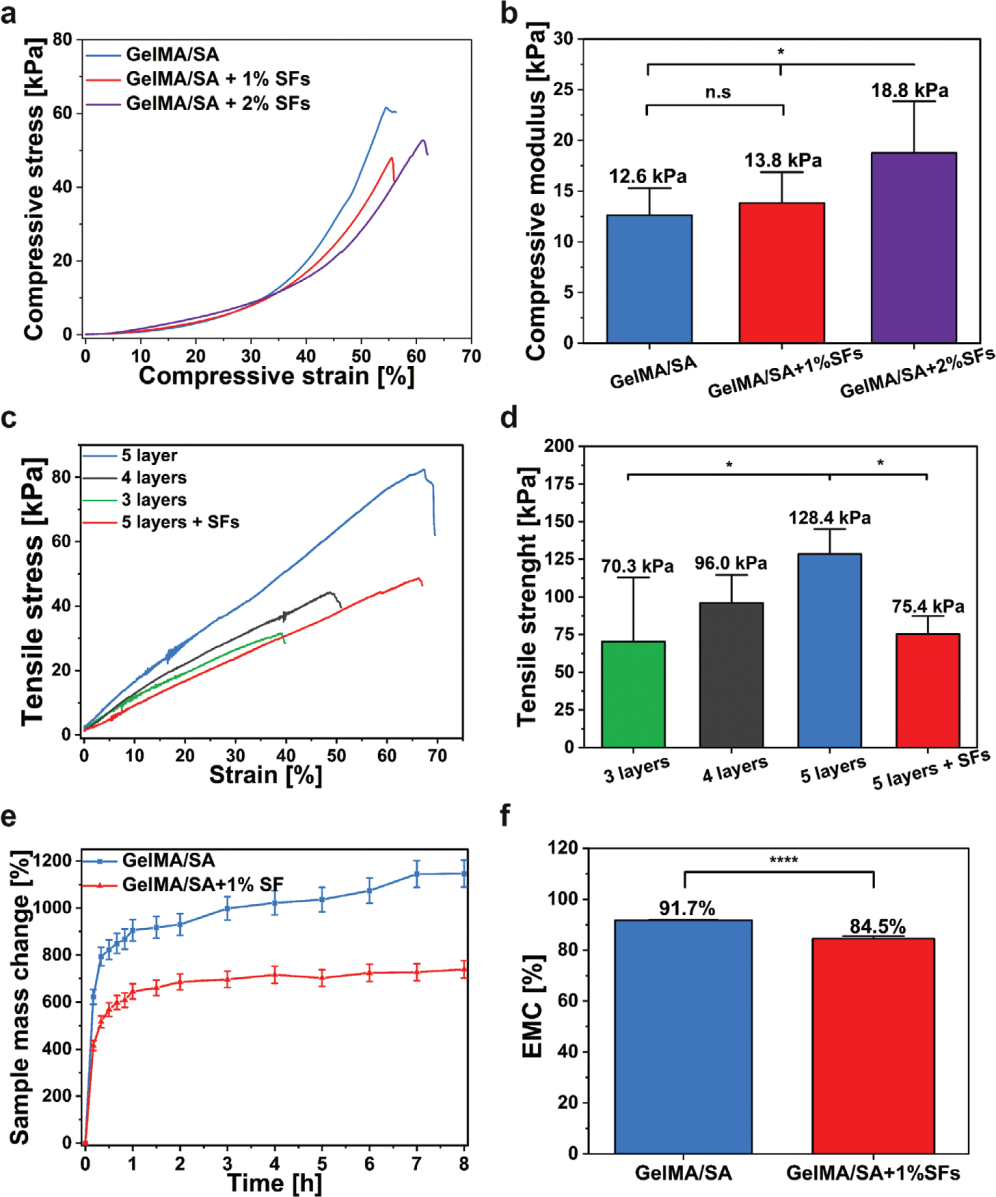
Physical characterization of the materials. a) Compressive stress‐strains curves of bulk hydrogels with different SFs concentrations and corresponding b) average compressive modulus. c) Tensile stress‐strain curves show the elongation of the 3D scaffolds with different printed layers, and the SFs' influence on this parameter shows the elongation at break and corresponding d) tensile strength. e) Sample mass change in time of 3D scaffolds and f) Equilibrium moisture content for the 3D print without and with 1% of SFs.

Hydrogels possess a mechanical strength, quantified by the compressive modulus, that falls within the 1–100 kPa range, lower than traditional wound dressings like gauze or cotton wool (1 MPa to 1 GPa).^[^ ^57^ ^]^ Nevertheless, this specific range of mechanical strength aligns better with the needs of human skin in terms of compatibility due to the similarity with soft tissues. Consequently, a key area of research focuses on developing highly effective hydrogel dressings with enhanced mechanical properties.^[^ ^58^ ^]^ The incorporation of SFs aimed to achieve the dual effect. On the one hand, SFs enhance the mechanical properties of the printed scaffold. On the other hand, they may decrease the swelling ratio due to the hydrophobic nature of PLGA polymer.^[^ ^33^ ^]^ The overarching goal was to balance mechanical strength and swelling behavior to preserve the dimensional fidelity of the printed structures. One of the most important features of such materials is their conformability to the skin tissue. The photos of printed material on human skin tissue were presented (Figure S3a, Supporting Information). The hydrogel demonstrated skin adhesiveness before and after bending, indicating the optimal properties for the application. Furthermore, the mechanical properties of the 3D printed scaffolds were evaluated by compression tests to assess the strength of hydrogels and the reinforcing effect of the incorporated SFs (**Figure** **3**a). Compression tests were conducted to analyze the compressive resistance of hydrogels and the impact of SFs on compressive strength and modulus (Figure 3a,b). The introduction of SFs into the network at the level of 1% does not significantly improve (p‐value ≤ 0.05, n = 3) the compressive strength (13.8 ± 3.1 kPa) compared to the pure hydrogel (12.6 ± 3.1 kPa). The increase of SFs concentration up to 2% significantly improves the compressive strength (18.8 kPa) compared to the 1% (*p* ≤ 0.05) and pure hydrogel (*p* ≤ 0.05) (Figure 3a). However, adding 2% of SFs poses challenges for printability due to the hydrophobicity of PLGA, which causes polymer agglomeration within the hydrophilic hydrogel network. The stress‐strain curves were obtained to investigate how the number of layers influences the critical strain (Figure 3c). The critical strain was 38%, 49%, and 66% for 3‐layered, 4‐layered, and 5‐layered prints, respectively. Moreover, the tensile strength for the same samples was 70.3, 96.0, and 128.4 kPa, respectively (Figure 3d). Nevertheless, adding 1% SFs to the 5‐layered structure decreased the tensile strength (75.4 kPa). Still, the critical strain was maintained at exactly (66%) the same level as for a 5‐layered structure without SFs. These results indicate that the mechanical strength of the scaffolds improved slightly with the addition of SFs and an increase in the number of printed layers.

### 2.4 Swelling Ratio, Equilibrium Moisture Content, and Water Retention Capacity

A critical parameter of hydrogels’ network is their capacity to absorb water, called swelling. The high water content supports hydrogels’ biocompatibility and permeability. It is a crucial parameter in determining the networks’ ability to transport tissue exudates, explaining their extensive use in wound healing applications.^[^ ^59^ ^]^ However, excessive water uptake can compromise the scaffolds’ initial shape and mechanical stability.^[^ ^60^ ^]^ The 3D scaffolds were tested in two configurations, GelMA/SA and GelMA/SA + 1% SFs, to evaluate SFs influence on the swelling ability. The scaffolds containing 2% SFs were omitted due to printability problems.

The results show that both samples reached the swelling equilibrium state quickly after 2h of the test (Figure 3e). During the test, the size and properties of scaffolds remained unchanged regardless of the surrounding environment. The GelMA/SA hydrogels absorbed a significant water amount (930 ± 46%), whereas the incorporation of 1% SFs significantly reduced the swelling by 245% (685 ± 35%). After 2 h of incubation, the water uptake notably slowed, showing a SR at 1146 ± 57% and 739 ± 37% for GelMA/SA and GelMA/SA + 1% SFs, respectively. Nevertheless, the hydrogels displayed stability over 8 h incubation time. The stability is attributed to the GelMA and SA successful cross‐linking process. In addition, the moisture content was calculated to determine the impact of SFs on the swelling ratio (SR) (Figure 3f). The hydrogels showed 91.7 ± 0.1% and 84.5 ± 0.7% of equilibrium moisture content (EMC) for GelMA/SA and GelMA/SA + 1% SFs, respectively. The results showed a significant decrease in water content comparing the plain bulk hydrogel with SFs incorporated (*p* < 0.0001). The results indicate that including hydrophobic SFs within the hydrogel matrix tends to decrease its hydrophilic nature and confirm that SFs reinforcement improves hydrogels’ properties.

The water retention capacity (WRC) test was conducted from equilibrium swollen GelMA/SA and GelMA/SA+ 1% SFs 3D prints to determine the hydrogel's dry matter and ability to maintain water content over time when placed at room temperature. As printed hydrogels, after 24h of incubation, and dehydrated, were photographed to define the volume change of 3D prints in different conditions (Figure S3b, Supporting Information). Material's surface area after 24h of incubation in 37 °C in HEPES increased from 1.25 to 1.35 cm^2^, indicating only 8% of surface area increase. Nevertheless, as it swelled, the hydrogels were subjected to the dehydration process. After 36h, the surface area significantly decreased to 0.174 cm^2^, showing an 87% decrease in surface area. The remaining weight of each sample during the WRC test was measured at predefined time points (Figure S3c, Supporting Information). The linear decrease of sample mass can be observed over the first 8h of the dehydration process, reaching equilibrium after 24h. The dehydration process is significantly slower for 3D prints containing SFs compared to the pure GelMa/SA hydrogel. However, no significant differences were observed in the final remaining weight for GelMA/SA (25.9 ± 10.6%) and GelMA/SA+1% SFs (32.5 ± 1.5%).

### 2.5 Photo‐Thermal Characterization of Scaffolds

**Figure 4:**
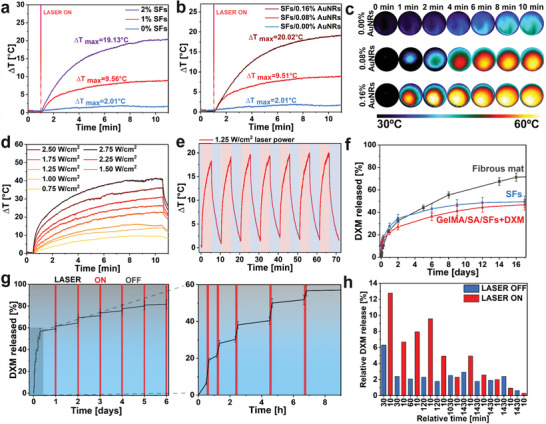
Photo‐thermal optimization of 3D printed material and drug release studies. a) Photo‐thermal properties comparison of the 3D printed material containing 0%, 1%, and 2% SFs. b) Photo‐thermal responsiveness of the 3D printing material containing 1% of SFs incorporated with 0.00%, 0.08%, and 0.16% of AuNRs. c) Panel with thermal camera images showing the reference 3D print without SFs/AuNRs and with SFs/AuNRs at 0.08% and 0.16% concentration. Sample temperature was shown before and after 10 min of NIR exposure. d) Temporal plots of different laser powers‐temperature dependence of 3D print over time using optimized 1% SFs/0.16% AuNRs concentration. e) Multiple NIR irradiation cycles demonstrate platform stability, with temperatures reaching consistent levels across every cycle and cooling down within 5 min after turning off the laser. f) Hydrogel influence on the DXM release profile. g) Gradual release of DXM under NIR laser irradiation from GelMA/SA+1%AuNRs/DXM 3D printed hydrogels. h) The graph quantifies DXM release at different intervals with and without NIR irradiation.

AuNRs show excellent NIR‐light absorption, generating a moderate temperature rise in the target region.^[^ ^61^ ^]^ The photo‐thermal effect provides temperature‐triggered and controlled drug release as well as bacterial eradication features. Thus, the response of the GelMA/SA + SFs/AuNRs scaffolds to NIR light was examined by irradiating them with a laser emitting an 808 nm wavelength beam. Different hydrogel configurations were tested (different SFs concentration and different AuNRs within SFs) to investigate the photo‐thermal properties of prepared composites. The maximum temperature the system can reach after 10 min of laser irradiation was recorded using a thermal camera setup. The scaffolds were optimized to reach the targeted temperature >55 °C (≈18 °C of ΔT starting from 37 °C). Different SFs concentrations (0%, 1%, and 2%) containing 0.08% w/w AuNRs were tested (**Figure** **4**a). The samples could be heated by 2.0, 9.6, and 19.1 °C for hydrogels containing 0%, 1%, and 2% of SFs, respectively. Considering the 2% concentration as sufficient for reaching the desired temperature but too high for printing application, we decided to double the AuNRs content to 0.16% w/w. The thermal behavior of the hydrogels containing different AuNRs concentrations (0%, 0.08%, and 0.16%) was evaluated by applying a constant laser power density of 1.50 W/cm^2^ (Figure 4b). Finally, as illustrated in the graph, a progressive increase in the Tmax was observed with increasing AuNRs concentration. The ΔT reached 20.12 °C for 0.16% w/w AuNRs content and 9.51 °C for 0.08% (w/w) (Figure 4c). According to the polymer Tg evaluation and considering the ideal temperature for photothermal therapy (PTT), the engineered material should reach 55 °C in order to eradicate bacteria and deliver the drug on demand.^[^ ^22^ ^]^ Consequently, an investigation into the maximum achievable temperature within the system was undertaken by employing a spectrum of different laser intensities (Figure 4d). The time‐temperature profiles were evaluated across a range of laser powers, starting from 0.75 W cm^−2^ up to 2.75 W cm^−2^. The investigation revealed a rapid and significant elevation in temperature that exhibited a direct proportionality to the applied laser power (Figure S4, Supporting Information). A robust linear correlation (R^2^ = 0.988) was established between the ΔT observed and the intensity of the laser light. Thus, the optimal parameters of NIR irradiation were selected as 1% SFs containing 0.16% wt AuNRs and 1.50 W cm^−2^. To ensure the consistency of the photo‐thermal response, the hydrogel was exposed to seven cycles encompassing 5 min of laser irradiation followed by 5 min of cooling phase (Figure 4e). Upon exposure to the laser beam, the material exhibited a rapid temperature increase, reaching a ΔT range between 18.5 and20.5 °C within 5 min. The temperature efficiently returned to its initial state after deactivation of the laser within the next 5 min. The cyclical process demonstrates the composite's excellent photo‐thermal conversion efficiency and rapid heat dissipation characteristics.

### 2.6 Drug Release Studies

**Figure 5:**
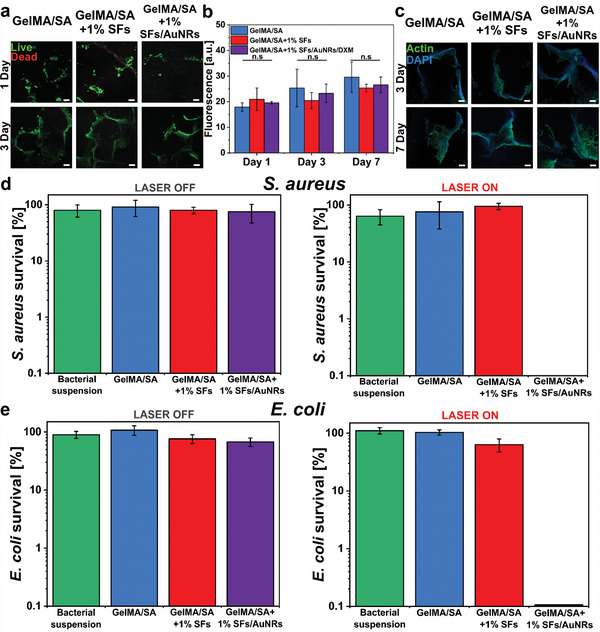
Direct cytotoxicity and antibacterial tests of GelMA/SA + 1% SFs/AuNRs, GelMA/SA + 1% SFs, GelMA/SA prints. a) Viability of L929 fibroblasts seeded on 3D printed structures. Live/Dead images captured on days 1 and 3 of culture: viable cells are stained green, while dead cells are marked red. Scale bar: 100 µm. b) Cell proliferation was assessed on days 1, 3, and 7, showing consistent cell increase over time in all tested samples. c) Morphological analysis of L929 fibroblasts seeded on printed structures at days 3 and 7. The actin cytoskeleton is stained green (Actin), while nuclei are stained blue (DAPI). Scale bar: 50 µm. Photothermal inactivation of d) *S. aureus* and e) *E. coli* via GelMA/SA+1%SFs/AuNRs material irradiated with NIR light. The survival percentage of bacteria was measured after 10 min for *E. coli* and 15 min for *S. aureus* or in the absence of irradiation (bacteria suspension was used as the control). Results are shown as mean ± standard deviation on a logarithmic scale.

The introduction of AuNRs and rapid temperature increase upon laser irradiation paved the way for on‐demand drug delivery applications. NIR stimulation, delivered directly to the injured site, triggers drug release within the therapeutic window, minimizing potential off target effects.^[^ ^21^ ^]^ This targeted and controlled delivery is particularly advantageous in chronic wounds, where prolonged inflammation hinders healing.^[^ ^62^ ^]^ The pulsatile release of drugs can be tailored to meet specific therapeutic needs. Light as a stimulus offers unique advantages, including high spatial and temporal precision, enhanced safety compared to traditional methods, and minimal disruption to cellular signaling pathways.^[^ ^63^ ^]^ We selected DXM as a glucocorticosteroid possessing anti‐inflammatory properties. The DXM release kinetics were evaluated before and after structurization into SFs. Four different temperatures (room temperature (RT), 37, 45, and 55 °C) were selected to understand temperature‐mediated release profiles and the influence of the heat produced by laser irradiation. To simulate the In vivo environment, the samples were positioned on a temperature‐controlled hot plate at 35 °C, corresponding to human physiological body temperature. Additionally, a higher temperature of 45 °C was employed, exceeding slightly the Tg of the incorporated SFs. This elevated temperature facilitates the acceleration of the release by promoting enhanced polymeric chain mobility. The 55 °C targeted temperature aligns with the established requirements for effective bacterial eradication using laser irradiation.^[^ ^65^ ^]^ The drug releases were conducted from as spun PLGA mat before SFs preparation (Figure S5, Supporting Information). The evaluation of cumulative DXM release over 14 days demonstrated a statistically significant correlation with temperature. The cumulative drug release at RT was 4%. This value increased to 73% at 37 °C. Further, an increase in temperature to 45 and 55 °C resulted in the cumulative release of 79% and 85%, respectively. No significant differences were found between higher 45 and 55 °C temperatures. On the first day of the test, 2%, 23%, 54%, and 56% of the drug is released from the matrix according to the increasing temperature. Higher temperatures resulted in a more significant initial burst of drug release. Further investigation explored how the material structurization into SFs impacted the release properties (Figure S6, Supporting Information). The exact temperature dependence resulted in 24%, 49%, 57%, and 66% DXM released after 14 days for RT, 37, 45, and 55 °C, respectively. Compared to the overall release, the release from the SFs‐containing material was 19% lower at high temperatures. This observation suggests a potential drug loss during the steps involved in material preparation. The results showed a promising approach for temperature‐controlled DXM release. Moreover, the influence of SFs incorporation into the GelMA/SA hydrogel was investigated (Figure 4f). However, no significant differences were found between the SFs before and after introduction into the hydrogel matrix in the initial phase (27% compared to 23%) and in cumulative release (49% compared to 47%) over 16 days. Nevertheless, a release was slightly more controlled between the 2nd and 12th days in the SFs incorporated hydrogel case compared to the SFs dispersed in a PBS medium. The release from the mat in the same conditions was included in the comparison to emphasize the advantage of SFs over the mat in controlling drug release. The 3D printed stimuli‐responsive system was subjected to cyclical laser irradiation during the core drug release test. Each cycle lasted 10 min of laser exposure, and the experiment comprised 11 cycles (Figure 4g). The sustained release of DXM at body temperature clearly distinguishes between the irradiated and non‐irradiated phases. In the initial 30‐min cycle, the release was about 6% (Figure 4h). However, during the first 10‐min laser irradiation, the release doubled to 12%. Over the first 5 cycles, more than twice the amount of DXM was released during laser exposure, even though the irradiation time was only 10 min. As the intervals between time points increased, the differences became less noticeable. After 1 day, the 10‐min irradiated phase released a similar amount of DXM as the non‐irradiated phase. In the final two cycles, as the drug concentration in the platform decreased and the release period extended, the irradiation phase released much less DXM than the non‐irradiated phase. Laser exposure accelerates drug release from the hydrogel, resulting in an almost double increase in cumulative release (81%) compared to the non‐irradiated samples in the body temperature condition (43%). This aligns with the data in **Figure** **5**a,b, demonstrating increased release at higher temperatures.

### 2.7 In Vitro Cell Studies

The biocompatibility of the proposed 3D printed constructs and their potential for biomedical uses and applications were assessed using L929 fibroblast cells. As reported in previous studies, the cells were seeded and cultured onto the 3D structures.^[^ ^66^, ^67^ ^]^ The presence of AuNRs and SFs in the hydrogel precursor was evaluated regarding cell response and cell‐materials interactions. Cell viability, proliferation, and morphology were evaluated and compared to 3D printed construct made of GelMA/SA. The viability was determined using a Live/Dead assay kit to stain in green color alive cells and red color dead cells. From the observation of representative images captured after Live/Dead staining, it is evident that the majority of cells are viable (green cells) at both time points (days 1 and 3) for all the tested samples (Figure 5a). Only a few dead cells (marked in red) are visible at the early stage of culture (day 1), and an even lower number of dead cells are present after 3 days of culture. This demonstrates the high viability of cells in contact with the proposed 3D printed structures and the cytocompatibility of all the components of the hydrogel precursor solution, including AuNRs, SFs, and GelMA/SA. No significant difference was detected among the selected conditions at any time point.

Figure 5b reports the graph of cell proliferation during the culture time (up to 7 days). Data show the linear increase in cell proliferation at each time point (1, 3, and 7 days) for GelMA/SA+1%SFs/AuNRs printed constructs and control 3D printed structures with no evident difference. On the other hand, cells cultured on filaments 3D printed samples do not display an increasing proliferation tendency on day 3 of culture, while the increment in cell proliferation is visible at day 7. At the latest stage of culture (day 7), all the tested conditions reported similar cell responses, highlighting the suitability of the 3D printed constructs for cell viability, metabolic activities, and proliferation.

Last, the cell morphology was evaluated by Actin/DAPI staining and subsequent imaging of the samples with a confocal microscope at days 3 and 7 of culture (Figure 5c). Cell cytoskeletons appeared adherent, spread, and elongated on the 3D printed fibers, adapting to the pores of the 3D printed constructs at each time point. The typical spindle shape of L929 fibroblasts is visible with no evident differences among the tested conditions, evidencing the efficient support of the proposed 3D printed structures for cell adhesion and spreading.

### 2.8 Evaluation of Antibacterial Properties

Discovering effective treatments for resistant gram‐positive and gram‐negative bacteria has been challenging due to their growing drug resistance.^[^ ^68^ ^]^ *S. aureus* and *E. coli* strains were chosen to assess the antibacterial properties of the prepared materials because they are the most common pathogens in infected wounds and are known for forming biofilms.^[^ ^69^ ^]^

The incorporated AuNRs demonstrated an exceptional temperature threshold for bacteria eradication after 10 min of NIR exposure (>55 °C) (Figure 4e,f). During the experiment, in the presence of GelMA/SA+1%SFs/AuNRs material, *S. aureus* strains were reduced to 3 log units (limit of detection), demonstrating bacterial survival below 0.1% (n = 3, Figure 5d). No colonies were also observed in the representative agar plates, effectively inactivating 99.95% of bacteria in the suspension (Figure S7a, Supporting Information). *E. coli* bacteria, when in contact with photo‐thermal material, were also reduced to 3 log units, resulting in only 0.1% survival (n = 3, Figure 5e). However, representative agar plates showed minimal bacterial colonies, indicating 99.90% bacterial inactivation (Figure S7b, Supporting Information). These findings confirm the effective antibacterial properties and bacteria eradication of GelMA/SA+1%SFs under NIR irradiation. Similar findings were presented in our previous work, where 10 min of laser exposure and a temperature threshold of 60 °C was sufficient to inactivate the *S. aureus* and *E. coli* to the limit of detection.^[^ ^40^ ^]^ However, photo‐responsive material without NIR light did not activate bacteria, nor did other tested materials under both irradiated and non‐irradiated conditions. The *S. aureus* and *E. coli* control suspensions were not inhibited with or without NIR light.

The results underscore the advantageous photo‐thermal properties of AuNRs for killing representative gram‐positive (*S. aureus*) and gram‐negative (*E. coli*) bacteria. While traditional light‐activated therapies promote bacterial growth inhibition, recent advancements have broadened their application to the induction of bacterial membrane disruption. Under NIR‐light, the composite material reached the temperature of 61.6 °C after 15 min, and 58.9 °C after 10 min of laser exposure during *S. aureus* and *E. coli* eradication, respectively, damaging bacterial membrane and causing cell death. A novel approach facilitates controlled drug release at the infection site, offering a promising strategy for combating bacterial wound infections. Furthermore, the photo‐thermal effect can enhance blood flow to the affected area, promoting natural wound healing.^[^ ^65^, ^70^ ^]^

### 2.9 In Vivo Healing Tests

**Figure 6:**
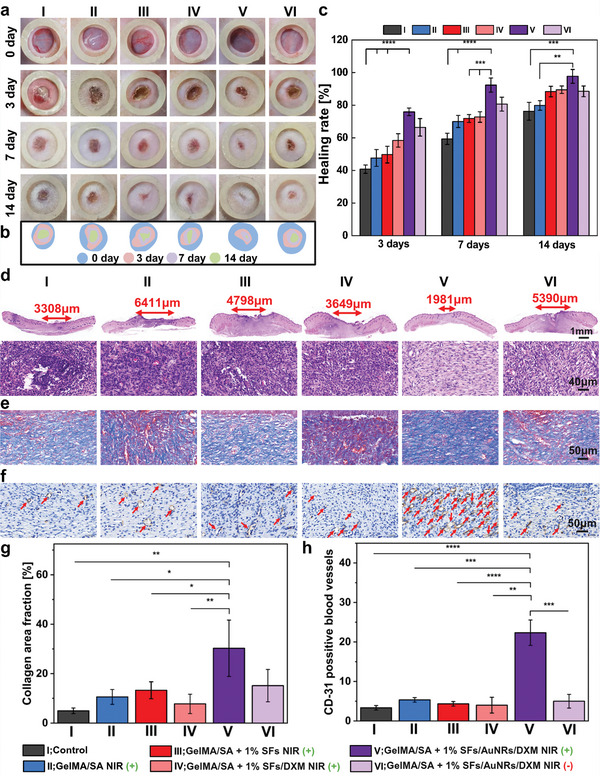
In vivo skin tissue healing of cutaneous and *S. aureus* infected wound on rats. a) Photographs of skin wounds on days 0, 3, 7, and 14 in the I – control group NIR (+), II – GelMA/SA NIR (+), III – GelMA/SA + 1% SFs NIR (+), IV – GelMA/SA+1% SFs/DXM NIR (+), V – GelMA/SA+1% SFs/AuNRs/DXM NIR (+), VI – GelMA/SA + 1% SFs/AuNRs/DXM NIR (‐) groups. b) The graphical visualization of the remaining wound area, and corresponding c) Healing rate of groups on 0, 3, 7, and 14 days. d) H&E staining results of the skin wound. The arrows show the edge and size of the immature granulated tissue. e) Masson's trichrome staining of infected wound tissue sections after 14 days of treatment. f) Representative images of CD31 immunohistochemistry staining of infected wounds after 14 days. The red arrows show new blood vascular networks. g) Quantitative collagen volume fraction corresponding to Masson's staining images quantified using ImageJ. h) Positive area of CD31 immunohistochemistry staining images showing the new blood vessel networks.

An infected rat wound model was established during animal experiments to verify the In vivo therapeutic effect of 3D printed GelMA/SA + 1% SFs/AuNRs/DXM scaffolds. A circular, full‐thickness skin defect was created on the back of rats (n = 4 per group), and *S. aureus* was added on the wound site to introduce bacterial infection. The *S. aureus* was selected as a model bacteria strain to test In vivo, as it is the most common pathogen of infected wounds. The wound size on the back of the rats after 3D printed scaffold treatment was continuously monitored at 0, 3, 7, and 14 days (**Figure** **6**a). It was macroscopically observed that the fastest healing appeared in group V. Moreover, each group materials were attached to the wound site and put under NIR laser treatment for bacteria eradication. The treatment time was 10 mins, and infrared thermographs of the hydrogels were taken at 0 and 5 min after laser exposure (Figure S8a, Supporting Information). Corresponding images of agar plates with bacterial colonies showed the bacteria survived after laser treatment. The antibacterial evaluation revealed that group V could reach 52.5 °C (ΔT = 20.8 °C) under the NIR laser after 5 min of irradiation. No photo‐thermal effect was found in other groups, as expected. An increase in temperature reduced the number of bacteria compared to the other groups where many bacterial strains were present, and a lower antibacterial effect was found. According to the results, the healing process of the GelMA/SA+1% SFs/AuNRs/DXM NIR (+) group was significantly accelerated. Simulated wound morphology (Figure 6b) showed that only the 2.3 ± 4.2% wound remained after 14 days of treatment in the main group (V). Compared to the groups where photo‐thermal effect did not appear (I, II, III, IV) or NIR treatment was not applied (VI), the 23.7 ± 5.5%, 20.1 ± 2.9%, 11.6% ± 3.2%, 10.5 ± 2.3%, and 11.5 ± 3.3% of the initial wound size remained, respectively. The healing rate of the GelMA/SA + 1% SFs/AuNRs/DXM NIR (+) group was the highest among all during entire treatment (75.9 ± 2.4% after 3 days, 92.3 ± 4.3% after 7 days, and 97.7 ± 4.2% after 14 days) (Figure 6c). After 7 days, the group was essentially cured, with healing rate at 92.3%. It was significantly more effective and faster in facilitating wound healing when compared to the control group (59.3%, p‐value ≤ 0.0001), pure hydrogel (70.0%, p‐value ≤ 0.0001), Gel‐MA/SA+1% SFs (71.9%, p‐value ≤ 0.001) and GelMA/SA+1% SFs/DXM (72.8%, p‐value ≤ 0.001) where the healing rates after 7 days of treatment were significantly lower. (Figure 6c). The H&E staining images demonstrated the granulation tissue and the newly formed epidermis (Figure 6d). Scars were present in the control, with immature granulated tissue seen in all groups. Group V exhibited the shortest immature granulation band (1981 µm), indicating the fastest healing, aligning with macroscopic observations compared to the control group I (3308 µm) and groups II (6411 µm), III (4796 µm), IV (3649 µm), and VI (5390 µm). Additionally, the photo‐thermal group treated with NIR light displayed new epidermis and dermal tissue with clear boundaries, along with hair follicles at the wound site. The Masson's staining and CD31 staining were conducted to visualize the new collagen fibers and vascularization in the wounds (Figure 6e,f). A quantitative evaluation showed that the treated group Gel‐MA/SA+1%SFs/AuNRs/DXM NIR (+) resulted in a significantly higher abundance of collagen fibers than the control (**p ≤ 0.01) and other groups without photo‐thermal agent (Figure 6g). The results showed that the collagen area fractions were 4.98%, 10.60%, 13.29%, 7.77%, 30.27%, and 15.17% positive testing area for control, I, II, III, IV, V, and VI group, respectively. A CD31 staining showed a greater presence of blood vascular networks and CD31 positive vessels (red arrows) in the GelMA/SA+1%SFs/AuNRs/DXM NIR (+) group compared to others. The statistical evaluation of CD31 positive blood vessels showed ≈85% (****p ≤ 0.0001) higher blood vessel expression in group V (22.33 vessel units) compared to the control (3.33 vessel units) (Figure 6h). Moreover, there were also significant differences between other groups – group II (5.33 vessel units; ***p ≤ 0.001), group III (4.33 vessel units; ****p ≤ 0.0001), group IV (4 vessel units; **p ≤ 0.01), and group VI (5 vessel units; ***p ≤ 0.001) compared to the group V. The results above indicated that photo‐thermal treatment improves the microenvironment balance by killing the bacteria and accelerating the wound healing process. Moreover, the H&E staining showed visibly lower inflammatory cell infiltration, so fewer inflammatory cells were present in group V than in the rest groups (Figure 6d). Thus, pro‐inflammatory cytokines such as TNF‐α and IL‐6 were marked in the wound tissues (Figure S8b,c, Supporting Information). The positive area analysis revealed a decreasing trend of TNF‐α and IL‐6 in the GelMA/SA+1% SFs/AuNRs/DXM NIR (+) group (Figure S8d,e, Supporting Information). The findings show significantly lower expression of TNF‐α and IL‐6 in all tested groups compared to the control group I. Moreover, the biggest difference is visible in the main group V, indicating that killing bacteria helps with microenvironment recovery, and on‐demand DXM delivery induce the anti‐inflammatory properties resulting in less inflammatory factor and cells. (***p ≤ 0.001).

In conclusion, treatment with GelMA/SA + 1% SFs/AuNRs/DXM hydrogel combined with NIR laser accelerated the wound healing process related to the photo‐thermal treatment and bacteria eradication, which resulted in a notable reduction in the wound area. Moreover, immunohistochemical analysis showed a higher abundance of collagen fibers, higher blood vessel expression, vascularization, and significantly less inflammatory reaction, resulting in lower TNF‐α and IL‐6 expression.

## 3 Conclusions

In this study, we successfully developed and characterized a novel photo‐responsive 3D printed scaffold using a composite of GelMA/SA hydrogel integrated with electrospun drug‐loaded SFs containing AuNRs. Incorporated SFs were photo‐thermally responsive due to the AuNRs presence and contained the anti‐inflammatory drug DXM. SFs were prepared using the cryocut technique, which produced uniform, non‐agglomerated fibers with low loss in the drug content that can be evenly dispersed within the hydrogel network. SFs slightly improved hydrogels’ mechanical properties. The scaffolds maintained structural integrity and mechanical strength, with higher tensile strength observed in multilayered constructs. The hydrogel demonstrated excellent water absorption capacity, essential for biocompatibility and permeability in wound healing applications. Integrating AuNRs within the SFs allowed for effective temperature increase via NIR light‐heat conversion. The scaffold exhibited a rapid temperature elevation, achieving the required thermal thresholds for bacterial eradication and controlled drug release. Repeated NIR treatments facilitated a controlled and gradual release of DXM. In vitro studies with L929 fibroblast cells showed high cell viability, proliferation, and appropriate morphology of cells seeded onto the scaffold. The photo‐responsive scaffold exhibited potent antibacterial properties against gram‐positive (*S. aureus*) and gram‐negative (*E. coli*) bacteria. NIR‐induced heating effectively reduced bacterial viability by over 99.9%. In vivo studies demonstrated that the hydrogel composite, combined with NIR treatment, facilitated infected wound healing. The scaffold promoted bacterial eradication, reduced inflammation, and induced vascularization, highlighting its therapeutic potential.

This is the first presentation of drug‐loaded photo‐responsive SFs reinforced into 3D print. We presented the mechanical approach for SFs production with low loss in active drug content while incorporating both AuNRs and stimuli‐responsive properties. This innovation opens new avenues for localized, controlled drug release in response to environmental stimuli, which is a significant advancement over existing materials. A major challenge in combining fibrous mats with hydrogels is the issue of delamination due to limited interfacial interactions between the hydrogel matrix and the fibers. In this work, we address this challenge by carefully optimizing the formulation and processing conditions, enhancing the compatibility between the fibers and the hydrogel matrix. This work contributes to the growing body of research on composite materials for tissue engineering and wound healing, offering a promising platform for advanced wound care. The scaffold's ability to provide structural support, controlled drug delivery, and potent antibacterial effects positions it as a versatile tool in regenerative medicine.

## 4 Experimental Section

### Materials

Poly(D, L‐lactide‐*co*‐glycolide) (PLGA, PDLG 5010, Corbion Purac) lactide: glycolide 50:50 (PLGA); 1,1,1,3,3,3‐ Hexafluoro‐2‐propanol (HFIP, Acros Organics); gold nanorods (AuNRs, λ = 810 nm, O.D. = 50, Au NR ≈ 1 mg mL^−1^, nanoComposix); Dexamethasone (DXM, >98% (HPLC) Sigma‐Aldrich); Tissue‐Tek O.C.T. compound (Sakura Finetek); Sodium alginate (SA, Alginic acid sodium salt from brown algae, low viscosity, Sigma‐Aldrich); Calcium chloride (CalCl_2_, Sigma‐Aldrich); 2‐Hydroxy‐4′‐(2‐hydroxyethoxy)‐2‐methylpropiophenone (Irgacure 2959, 98%, Sigma‐Aldrich); Gelatine type A (Sigma‐Aldrich); Phosphate buffered saline (PBS, pH ≈7.4, Sigma‐Aldrich); Methacrylic anhydride (2000 ppm topanol A as inhibitor, 94%, Carl Roth); HEPES (>99.5%, Carl Roth); hexamethyldisilazane (HMDS, 99.0%, Sigma‐Aldrich); Bovine serum albumin (BSA) (Sigma‐Aldrich); phosphate buffer saline (PBS, Sigma‐Aldrich); Triton X (Sigma‐Aldrich); DAPI (Sigma‐Aldrich); L929 murine fibroblasts (Sigma‐Aldrich); Dulbecco's modified Eagle's medium (DMEM, Gibco Invitrogen); fetal bovine serum (FBS, Gibco Invitro‐gen); penicillin streptomycin (PS, Gibco Invitrogen); EDTA‐trypsin (Gibco Invitrogen); Alexa Fluor 488 Phalloidin (Thermo‐Fisher Scientific); Live/Dead assay (Thermo‐Fisher Scientific); Haematoxylin‐Eosin Staining kit (Jiancheng Bioengineering Institute); Masson's Trichrome Staining Kit (Solaibio); Anti‐IL‐6 Rabbit pAb (BioVision); Anti‐TNF‐α Rabbit pAb (TNF‐α, Abcam); Anti‐CD31 Rabbit pAb (Cell Signaling Technology).

### Preparation of Electrospun Short Filaments

9.5% (w/w) PLGA in HFIP was prepared as an electrospinning polymer solution. The DXM was dissolved in the solution at a concentration of 10% (w/w) concerning the polymer weight. Additionally, an AuNRs suspension was mixed with the polymer precursor and gently stirred until a homogeneous solution was obtained (0.16% (w/w) to the polymer weight). The electrospinning was applied to fabricate an aligned PLGA mat. The positive voltage of 15 kV was applied to the 24G cut and blunt needle. The solution flow rate was set at 800 µL h^−1^ and the fibers were collected on a drum collector, rotating at 2500 rpm. The distance from the end of the needle to the collector was set to 15 cm. The room temperature and 50–60% relative humidity were maintained throughout the whole process. PLGA mats without DXM and AuNRs were fabricated under identical conditions as a control. Aligned polymer mats were cut into 1 cm x 2 cm rectangles, embedded in Tissue‐Tek, frozen, and cryo‐sectioned perpendicularly into 10 µm‐thick strips using a cryostat. The strips were homogenized at 15,000 rpm for 5 min and washed at least three times with distilled water via centrifugation to isolate the SFs.

### Preparation of the 3D Printed Hydrogel

GelMA synthesis involved reacting gelatin with methacrylic anhydride. A 10% (w/v) gelatin solution was prepared by dissolving gelatin in phosphate buffer (pH 7.5) at 50 °C with continuous stirring at 240 rpm. Once fully dissolved, methacrylic anhydride (0.5 mL per gram of gelatine) was added dropwise at a rate of 0.5 mL min^−1^ under vigorous stirring while shielding the reaction from light. After 3h, the mixture was diluted with warm PBS, dialyzed against distilled water (MWCO = 12–14 kDa) at room temperature for 10 days, and subsequently freeze‐dried.

The GelMA‐SA/SF printing inks were prepared by dissolving lyophilized GelMA and SA in 25 mM HEPES solution and mechanically stirred for 3 h at 44 °C. The SFs powder was mixed with ink, and just before the printing process, the 0.1% Irgacure 2959 as a photo‐cross‐linker was added. The final ink consisted of 6%–3.5%/1% GelMA‐SA/SFs, respectively.

Prepared inks were transferred to the UV‐protected syringes (to avoid photo‐cross‐linking of ink). The scaffolds were fabricated layer by layer using an extrusion‐based 3D bioprinter (CELLINK INKREDIBLE+) through a tapered metal nozzle (27G) with the printing speed set to 16 mm s^−1^. The printing pressure was set to 110 kPa, and the temperature was maintained at 22–23 °C. The hydrogel was cured with UV light and sprayed with 1% (w/w) calcium chloride solution during printing. After the preparation, the scaffold was cured with high‐power UV light (Dymax, 400 W, power density of 225 mW cm^−2^) and then immersed in 2% (w/w) calcium chloride for 10 min. Washed three times with deionized water and stored in HEPES solution.

### Physicochemical Characterization

DSC analysis was employed to examine polymeric nanofibers' glass transition temperature (Tg) and to understand how their structure affects this property. A PYRIS‐1 DSC PerkingElmer calorimeter was employed to acquire the measurements, and OriginPro software was used to analyze the data. The samples were subjected to a temperature range of 0 to 100 °C at a heating rate of 10 °C per minute, with each sample weighing ≈2.5 mg. The intersection of tangents of the cooling curves method in OriginPro software was used to determine Tg.

FTIR spectroscopy was used to analyze chemical bonds and confirm the chemical structure of both the polymeric material and the drug after maintaining structurization. FTIR spectra were acquired using VERTEX 70 (Bruker) equipment, scanning between 4000 and 400 cm⁻¹ with a resolution of 2 cm⁻¹. Prior to analysis, the cross‐linked hydrogel samples were lyophilized. Spectra were recorded for the PLGA mat, PLGA/SFs containing DXM, and dry DXM powder.

The scanning electron microscope (SEM, JSM‐6010PLUS/LV, In TouchScope microscope) and field emission scanning electron microscope (FE‐SEM, ZEISS Crossbeam 350 FIB‐SEM microscope) were utilized to observe the morphological characteristics of the bulk hydrogels and scaffolds. The hydrogels were synthesized following the previously outlined 3D printing ink preparation procedure. Bulk hydrogels underwent a process of cross‐linking, washing, and reaching a state of equilibrium through swelling at a temperature of 37 °C, after which they were subjected to freeze‐drying in a lyophilizer for 24 h. As for the 3D printed scaffolds, they were cross‐linked, washed and dehydrated. The samples underwent dehydration using a series of ethanol solutions with progressively increasing concentrations (20%, 40%, 50%, 70%, 80%, 90%, and 100%), followed by increasing graded HMDS in ethanol (20%, 40%, 50%, 70%, 80%, 90%, and 100% HMDS). A gentle dehydration process is very important to protect its organized structure which can be destroyed during lyophilization.^36^ After that, scaffolds were dried overnight at RT. Subsequently, the bulk and 3D scaffold hydrogels were coated with a layer of gold and visualized using the SEM and FE‐SEM.

A transmission electron microscope (TEM) (TALOS F200X, Thermo Fischer Scientific with field emission gun (X‐FEG)) was used to examine the AuNRs's existence and dispersion within the fiber structure after electrospinning. The fibers were deposited on mesh copper formvar grids by direct electrospinning.

The shape fidelity of 3D prints was evaluated based on qualitative macroscopic images, followed by the computation of a printability index (PI). The macroscopic images from each experimental group were analyzed using the ImageJ software, enabling the measurement of the perimeter and area of the interconnection channels (n > 20). Subsequently, the PI value was determined by normalizing the pore perimeter concerning the pore area using the equation S1, Supporting Information.

In the presence of optimal gelation conditions or when exhibiting ideal printing suitability, the affirmative channel of the structural entity assumes a square shape, resulting in a Pr value of 1 (excellent uniformity). However, a Pr value exceeding 1.1 indicates inadequate, unacceptable uniformity.

### Mechanical Tests

Compression tests were conducted on bulk hydrogels with dimensions of 11.2 mm in diameter and 15 mm in height using a DCX Texture Analyzer (AMETEK Brookfield, USA) at a constant speed of 10 mm min^−1^. During the static compression test, samples were compressed to failure. The compressive modulus was calculated from the slope of the stress‐strain curve at 10% strain, while the compressive strength was defined as the stress at the fracture point. Linear regression was applied to the stress‐strain curve between 10% and 15% strain to determine the compressive modulus. Each mechanical test was repeated three times on separate samples to ensure reproducibility.

To examine the suitability of 3D printed scaffolds for wound dressing, the mechanical properties of 3D scaffolds with and without short fibers were evaluated through a tensile test. The test used a CTX texture analyzer (AMETEK Brookfield, US) with a 10 N load cell. The analyzer was equipped with custom handles designed for delicate samples. Rectangular samples with 10 × 40 mm dimensions were securely placed during the test. The thickness of the 3D scaffolds was measured with Kroeplin calipers for each sample four times and then averaged for further analysis. The crosshead speed was set at 0.5 mm s^−1^. By analyzing the stress‐strain curves obtained from the test, essential mechanical parameters such as tensile strength and elongation at break were determined. Each formulation was subjected to three repetitions, and the tests were performed on as printed samples under ambient conditions.

### Equilibrium Moisture Content, Swelling Ratio, and Water Retention Capacity

The EMC was evaluated to measure the 3D scaffold's hydration activity. Briefly, the samples were fully immersed in HEPES solution until the equilibrium. Samples were taken out, and the moisture on the surface was gently wiped off. The samples were dried at RT until stable quality was reached (n = 4). EMC was calculated using the equation S2, Supporting Information.

Dynamic swelling experiments were conducted by immersing desiccated printed constructs in HEPES solution and monitoring their weight gain over time. The samples underwent dehydration using a series of ethanol solutions with progressively increasing concentrations (20%, 40%, 50%, 70%, 80%, 90%, and 100%), followed by air drying at RT. The dried scaffolds were then immersed in 3 ml of HEPES solution at 37 °C and allowed to reach equilibrium (n = 4). The mass of the scaffolds was measured at predetermined time intervals. The SR was calculated using equation S3, Supporting Information.

Swelled hydrogel prints without SFs and containing SFs were placed at room temperature and 40% humidity to examine the water retention percentage of hydrogel samples at constant conditions. Each sample (n = 5) was weighted at predefined time points (1, 2, 3, 4, 5, 6, 7, 8, 24, and 36h). The photos of swelled hydrogel and after dehydration were taken. The WRC was calculated using the equation S4, Supporting Information.

### Photo‐Thermal Characterization

The temperature variations of the materials before and after their transformation into short filaments and the printed composites were analyzed under NIR irradiation using an infrared thermal imaging camera (FLIR A655sc). Thermal characterization was performed on both dry and wet material states, and the relationship between NIR laser power and ΔT was examined. An 808 nm NIR laser was applied to test different AuNRs concentrations over a fixed duration. Initially, a concentration of 0.06% (w/w) AuNRs was tested, and based on the results, it was increased to 0.16% (w/w) for further analysis. Temperature measurements were recorded at 1‐s intervals using the FLIR Studio software setup.

### In Vitro Drug Release Studies and Release Kinetics

DXM release studies were conducted on electrospun materials both before and after structurization into short filaments, as well as on 3D‐printed porous scaffolds. Unstructured electrospun mats (≈10 mg) were cut into rectangular sections and immersed in 1 mL HEPES buffer (pH 7.4). Samples were collected at predefined intervals: initially at 0.5, 1, 2, 4, 6, 24, and 48 h, followed by every 3 days up to day 18. Similarly, ≈10 mg of dried short filaments were placed in the same release medium, and after centrifugation at 15,000 rpm for 3 min, the solutions were collected. The 3D‐printed scaffolds were prepared and washed following the same protocol and subjected to release studies in HEPES buffer. The effect of temperature on DXM release kinetics was assessed at RT, 37, 46, and 56 °C. To simulate skin conditions at RT, samples were placed in 2 mL vials on a glass hot plate maintained at 35 °C. Additionally, photo‐responsive samples were exposed to NIR laser irradiation for 15 min at specified intervals. Each release condition was tested in triplicate for accuracy. DXM concentrations at each time point were quantified using a UV spectrophotometer (Multiskan GO, Thermo Scientific) at the maximum absorption wavelength of 241 nm, which was determined using a DXM standard. Final DXM concentrations were calculated using a pre‐established calibration curve. Measurements for the polymeric material were compared against blank HEPES controls, while measurements for bulk hydrogels/SFs were normalized against hydrogel reference solutions (hydrogels without DXM) to minimize interference from hydrogel components.

### In Vitro Cell Studies

L929 fibroblast cells were cultured in DMEM modified with 10% FBS and 1% PS. Cells were seeded on a culture dish and incubated at 37 °C and 5% CO_2_. Every other day, the culture medium was refreshed. At ≈80% of confluence, cells were harvested from the culture plate by a first wash in PBS and subsequent incubation in 0.05% EDTA–trypsin at 37 °C and 5% CO_2_ for 3 min. Cells were centrifuged in a falcon tube at 1200 rpm for 5 min. The pellet formed after centrifuging was resuspended in a culture medium using a calculated volume to obtain the desired cell density.

3D printed scaffolds were produced upon sterilization of the polymer powders and filaments under UV light (30 min). Additionally, the filtration of the HEPES and CaCl_2_ solutions was performed using 0.22 µm filters. After fabrication, the 3D printed scaffolds were sterilized under UV light for 30 min and then placed in a 1.5 mL sterile Eppendorf. For seeding, cells were detached from the culture plate and diluted in 1 million cells mL^−1^ suspension. Then, 0.5 mL of cell suspension (equal to 0.5 million cells) was added to each Eppendorf and incubated at 37 °C and 5% CO_2_ for 30 min. Afterward, scaffolds and cell suspension were transferred in a non‐adherent well‐plate (Corning® Costar® Ultra‐Low Attachment Multiple Well Plate) and 4 mL of fresh culture medium was added. Samples were cultured in an incubator at 37 °C and 5% CO_2_ for 7 days.

Cell viability was investigated after 1 and 3 days of culture using a live/dead assay staining kit. Briefly, cells were washed with PBS and stained using a solution made of 0.5 µL mL^−1^ calcein (green staining of live cells) and 2 µL mL^−1^ ethidium homodimer (red staining of dead cells). Samples were incubated for 10 min at 37 °C and 5% CO_2_. Samples were washed trice with PBS and imaged using a confocal microscope (Leica TCS).

Cell proliferation after 1, 3, and 7 days of culture was assessed using the PrestoBlue assay on five replicates. At each time point, samples were treated with a 10% (v/v) PrestoBlue dilution in culture medium and incubated at 37 °C and 5% CO_2_ for 1 h. After incubation, 100 µL aliquots of the PrestoBlue reagent were collected and transferred to a 96‐well plate. The solution was then analyzed with a fluorometer – 530 nm excitation, 620 nm emission. (Fluoroskan Ascent TM Microplate Fluorometer, Thermo Scientific).

Cells morphology was observed using a confocal microscope (Leica TCS). Samples were washed with PBS and fixed at RT in 4% paraformaldehyde for 30 min. After washing with PBS, cells were incubated with 0.3% (v/v) Triton X‐100 for 15 min. Then, samples were treated with 1% (w/v) BSA solution prepared in PBS for 30 min and incubated in 1: 40 Alexa Fluor 488 Phalloidin in PBS for 40 min. Samples were protected from light with aluminum foil. Cell nuclei were stained with 1: 500 DAPI solution in PBS for 10 min. Samples were washed three times and finally imaged with a confocal microscope.

### Culture and Isolation of Bacterial Colonies

Colonies of *Escherichia coli* (ATCC 25922) and *Staphylococcus aureus* (ATCC 6538) were grown on lysogeny broth (LB) agar plates. The streak plate method was used to isolate bacterial colonies, which were inoculated in 3 mL of fresh LB broth and incubated in an orbital shaker at 37 °C for 16 h. Prior to testing, the 3D‐printed samples were sterilized under UV light for 30 min, while the polymer powders were sterilized by UV irradiation and HEPES/CaCl₂ solutions were filtered through 0.22 µm membranes. Three replicates of each sample were placed in sterile 96‐well plates for NIR irradiation testing. A single colony of *E. coli* or *S. aureus* was inoculated overnight in LB broth and diluted the following day with sterile PBS to achieve a bacterial concentration of 10⁶ CFU mL^−1^. 100 µL of *E. coli* suspension and 75 µL of *S. aureus* suspension were added to the samples' wells for testing. After optimizing the experimental parameters, samples were exposed to NIR laser irradiation for 10 min for *E. coli* and 15 min for *S. aureus*. Control samples (not irradiated) were incubated at RT in the dark under identical conditions. Post‐irradiation, 100 µL of sterile PBS was added to each well, and the bacterial suspensions were resuspended, serially diluted, and transferred to fresh 96‐well plates. Triplicate 5 µL aliquots of each dilution were plated on LB agar and incubated overnight at 37 °C. Bacterial survival was determined by colony counting. The maximum detection limit of the plating method was 3 log CFU mL^−1^, allowing for a minimum survival rate detection of ≥0.1%. Additionally, bacterial suspensions were further diluted 10‐fold for macroscopic visualization of survival at 0.05%. Diluted samples were evenly spread onto LB agar plates, incubated overnight, and photographed for analysis.

### In Vivo Experiments

All the In vivo experiments were conducted following the procedures approved by the Institutional Animal Care and Use Committee of Army Medical University (AMUWEC20232435). SD rats of 6–8 weeks were selected for the experiment. After depilation on the back of rats, a wound with a diameter of 1 cm was created using a punch, and a solution of Staphylococcus aureus was dripped onto the wound. Subsequently, various 3D printed hydrogel scaffolds were placed on the wounds of SD rats, and NIR irradiation (808 nm, 2 W) or no irradiation was performed for 10 min. Afterward, the wounds were photographed at different time points (0, 3, 7, and 14 days). The wound area was measured and analyzed using ImageJ software, and the healing rate was calculated. At last, the rats were sacrificed, and the skin tissues were collected, embedded in paraffin, and sectioned. H&E, Masson, and immunohistochemical staining were performed to analyze and evaluate the wound repair effect. The quantitative and statistical analyses were performed using ImageJ.

### Statistical Analysis

All tests were performed in at least triplicate (n > 3). Data are presented as mean ± standard deviation (SD). Statistical analysis was conducted using one‐way analysis of variance (ANOVA), followed by Tukey's post‐hoc test to assess significant differences. Results were considered statistically significant at the following p‐values: *p ≤ 0.05, **p ≤ 0.01, ***p ≤ 0.001, and **p ≤ 0.0001.

## Conflict of Interest

The authors declare no conflict of interest

## Author Contributions

D.R. and J.D. contributed equally to this work. designed experiments and performed physicochemical experiments, in vitro drug release studies, and swelling studies, analyzed data, wrote the manuscript, and prepared graphical illustrations; J.D. performed material characterization and wrote and edited the manuscript, prepared graphical illustrations; P.N. performed mechanical experiments and edited the manuscript; C.R. performed in vitro cell study and in vitro antibacterial test; A.K.K. prepared materials and supervised studies; A.Z. performed FE‐SEM imaging; H.W performed in vivo studies, analyzed and evaluated data; J.L. performed in vivo studies, analyzed and evaluated data; X.L. edited manuscript, and supervised experiments; B.D. edited manuscript, supervised experiments, and project; Y.Y. edited manuscript, supervised in vivo experiments and project; F.P. edited the manuscript, designed and supervised the experiments and project; all authors have approved the final version of the manuscript.

## Supporting information



Supporting Information

## Data Availability

The data that support the findings of this study are available from the corresponding author upon reasonable request.
