# Supplementary material for: NIR‐Light Activable 3D Printed Platform Nanoarchitectured with Electrospun Plasmonic Filaments for On Demand Treatment of Infected Wounds
Source: Adv Healthc Mater. 2024 Dec 25;14(6):2404274. doi: 10.1002/adhm.202404274 (PMC11874648; doi:10.1002/adhm.202404274)
Supplement: Supplementary file 1 — Supporting Information [file ADHM-14-0-s001.docx]

[Supporting Information]

NIR-Light Activable 3D-Printed Platform Nanoarchitectured with Electrospun Plasmonic Filaments for On-Demand Treatment of Infected Wounds

*Daniel Rybak,^1‡^ Jingtao Du,^2‡^ Paweł Nakielski,^1^ Chiara Rinoldi,^1^ Alicja Kosik-Kozioł,^1^ Anna Zakrzewska,^1^ Haoyang Wu,^3^ Jing Li,^3^ Xiaoran Li,^2^ Yunlong Yu,^3*^ Bin Ding,^2*^ and Filippo Pierini^1*^*

^1^Department of Biosystems and Soft Matter, Institute of Fundamental Technological Research, Polish Academy of Sciences, Warsaw 02-106, Poland

^2^Innovation Center for Textile Science and Technology, College of Textiles, Donghua University, Shanghai 201620, PR China

^3^Institute of Burn Research, Southwest Hospital, Third Military Medical University (Army Medical University), Chongqing 400038, PR China

^‡^These authors contributed equally to the work.

*Corresponding authors’ E-mail addresses: fpierini@ippt.pan.pl, binding@dhu.edu.cn, yuyunlong@tmmu.edu.cn.

Eqn. S1:

$$PI=L^{2}/16A$$

Where L is the perimeter, and A is the area.

Eqn. S2:

$$EMC=(mwet-mdry)/mdry*100\%$$

Eqn. S3:

$$SR=(mwet-mdry)/mdry*100\%$$

Where m*wet* and m*dry* represent the wet and dry weights of the samples, respectively.

Eqn. S4:

$$WRC=(msw-mdry)/mdry*100\%$$

Where msw represents the weight of swollen sample, and mdry the weight of dry hydrogel at each time point.


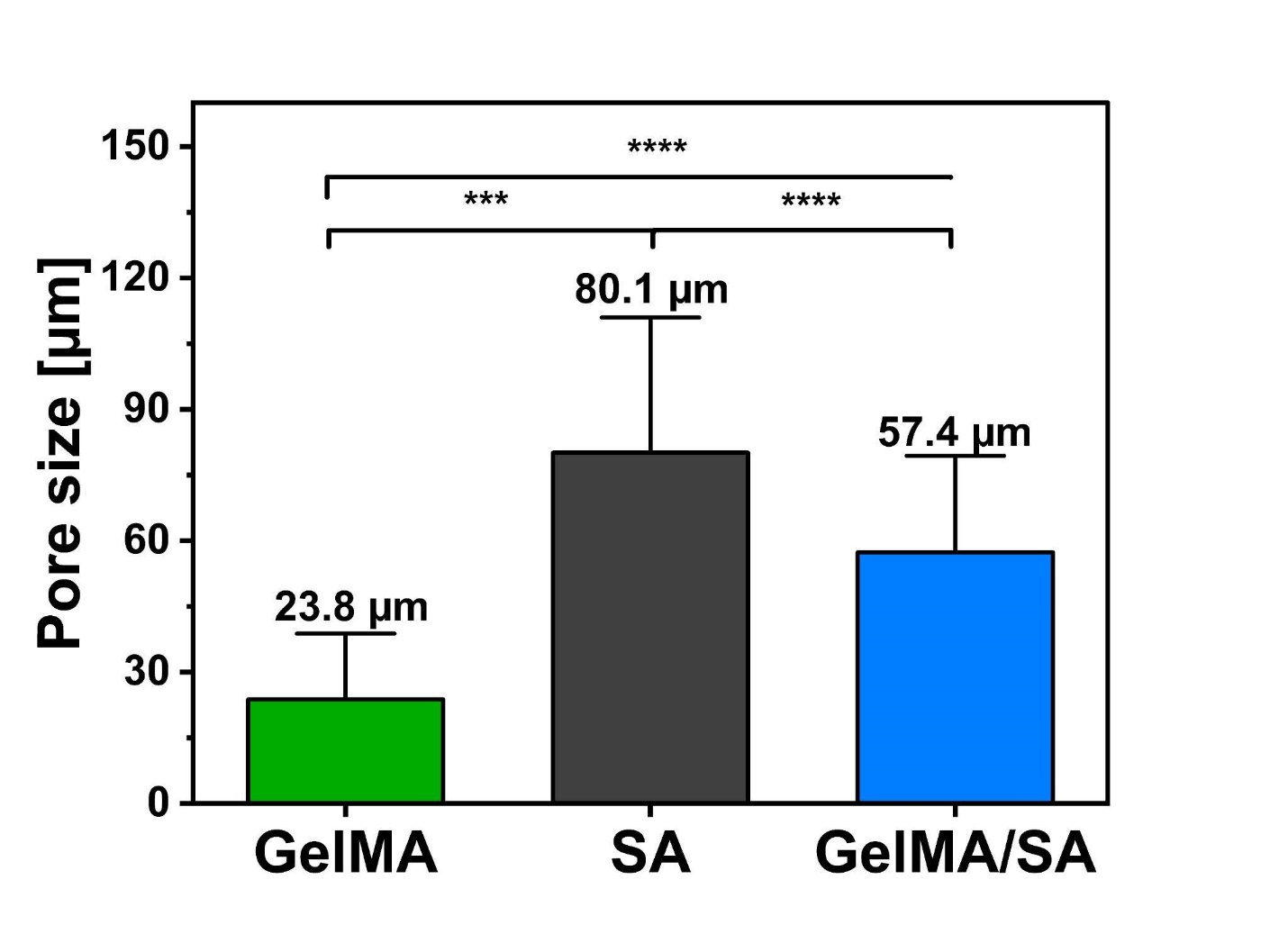
**Figure S1.** Average pore size (n = 20) for hydrogel components and GelMA/SA composite calculated from SEM images.


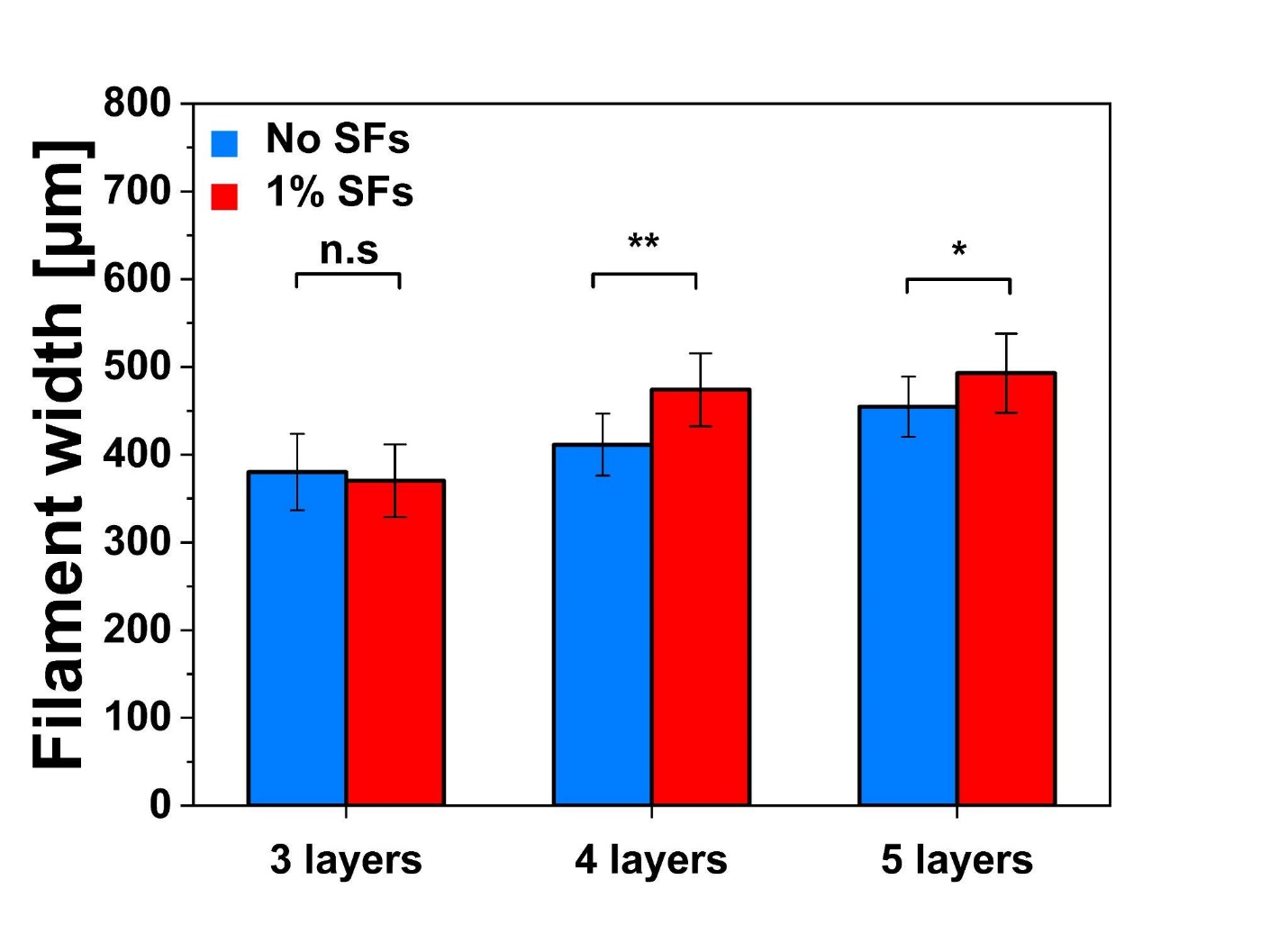
**Figure S2.** Filament lines width of 3D-printed scaffolds by inks with no SFs and with 1% of SFs with different layers.


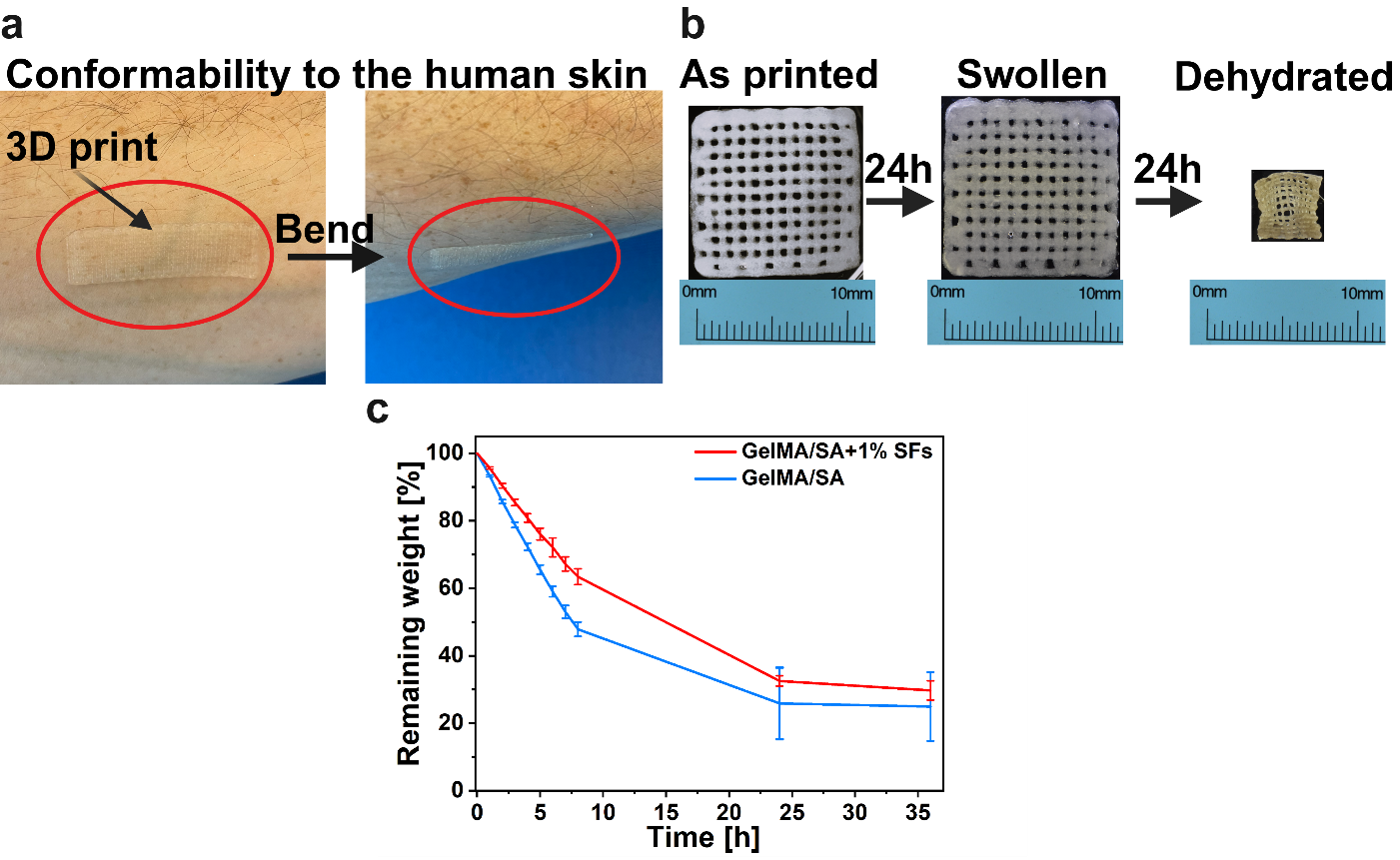


**Figure S3.** Additional physical hydrogel characterization. a) Conformability of the hydrogel to the human skin before and after bending. Hydrogels’ WRC – b) photos of as printed hydrogel, swelled hydrogel after 24 h of incubation, and dehydrated after 24 h. c) WRC showing the dehydration profile of GelMA/SA 3D prints with and without SFs.


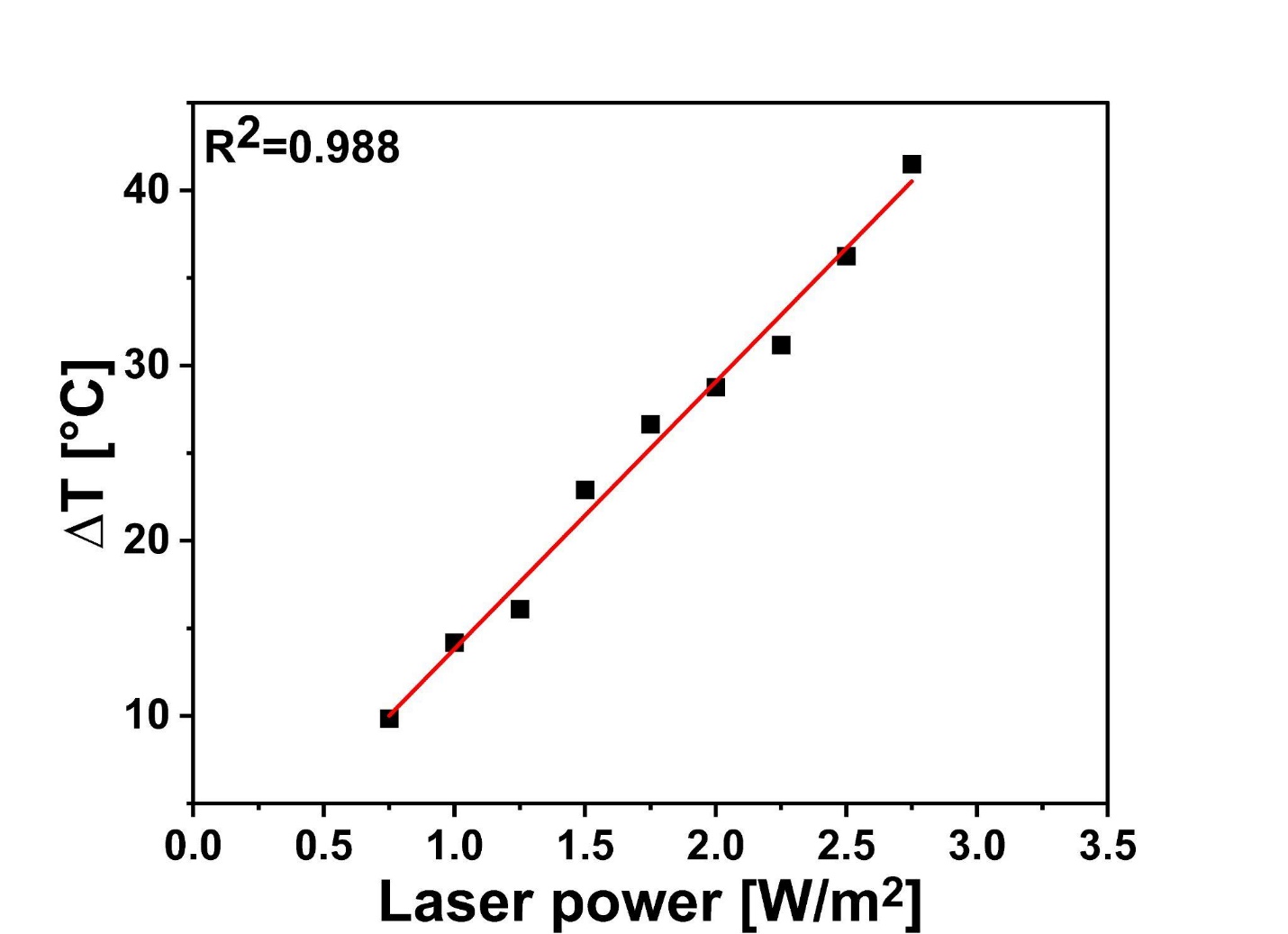
**Figure S4.** Linear plot of laser power and ΔTemperature dependence.


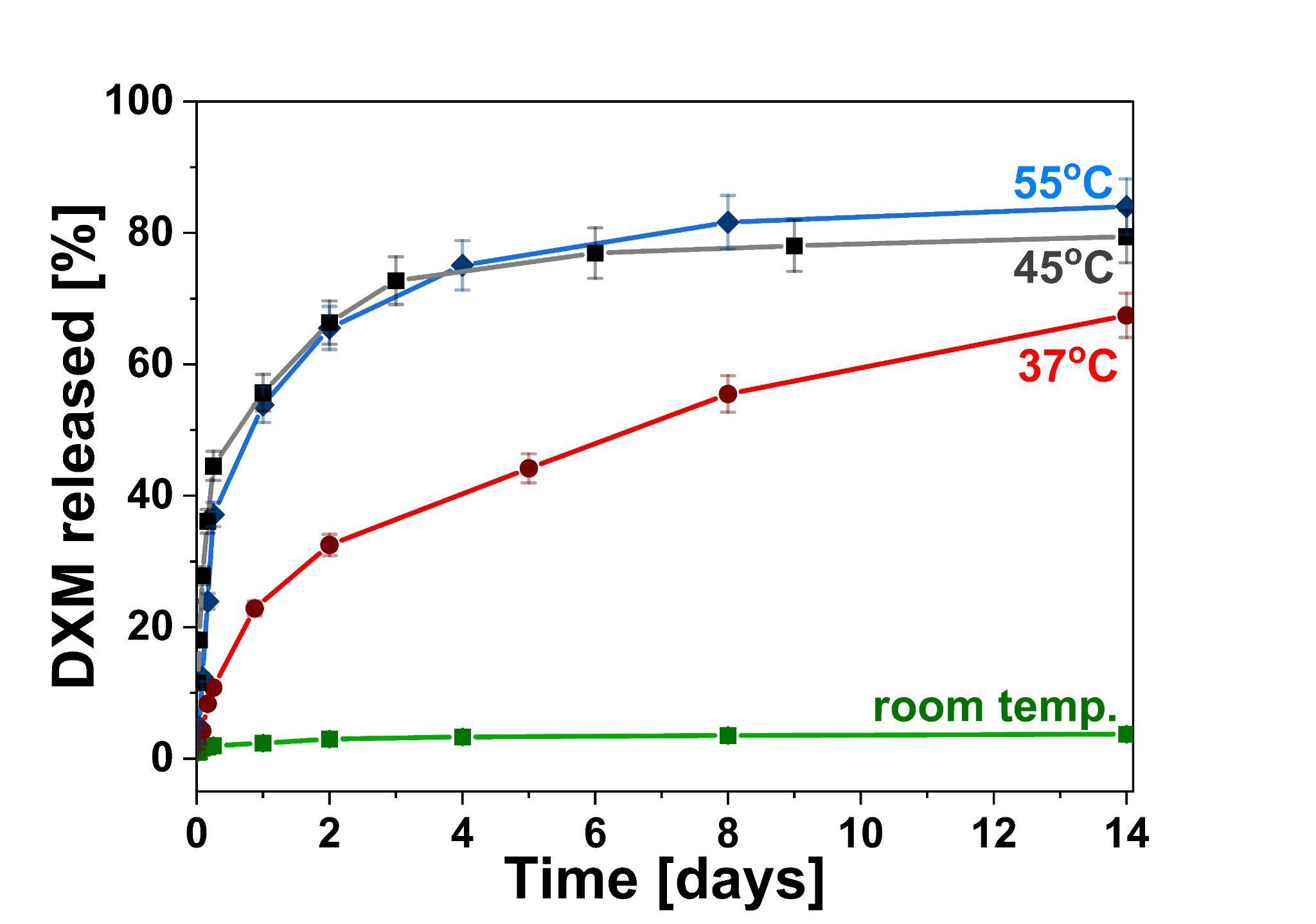
**Figure S5.** Drug release profiles from fibrous mats before structurization into SFs show the evident release kinetics depending on the temperature – higher temperature increases the release speed from the matrix.


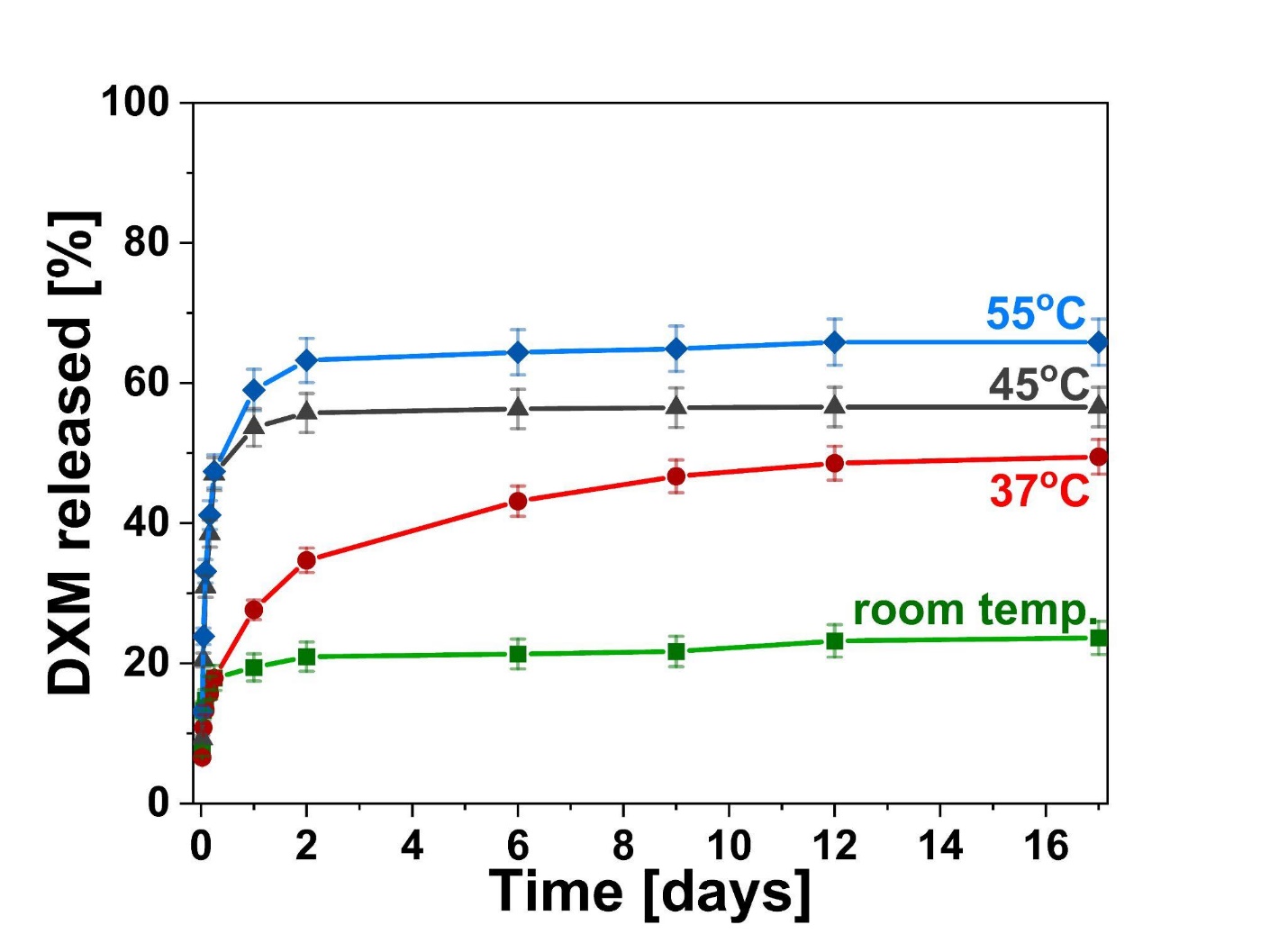
**Figure S6.** Drug release profiles from SFs after structurization; the same temperature dependence is maintained as before structurization.


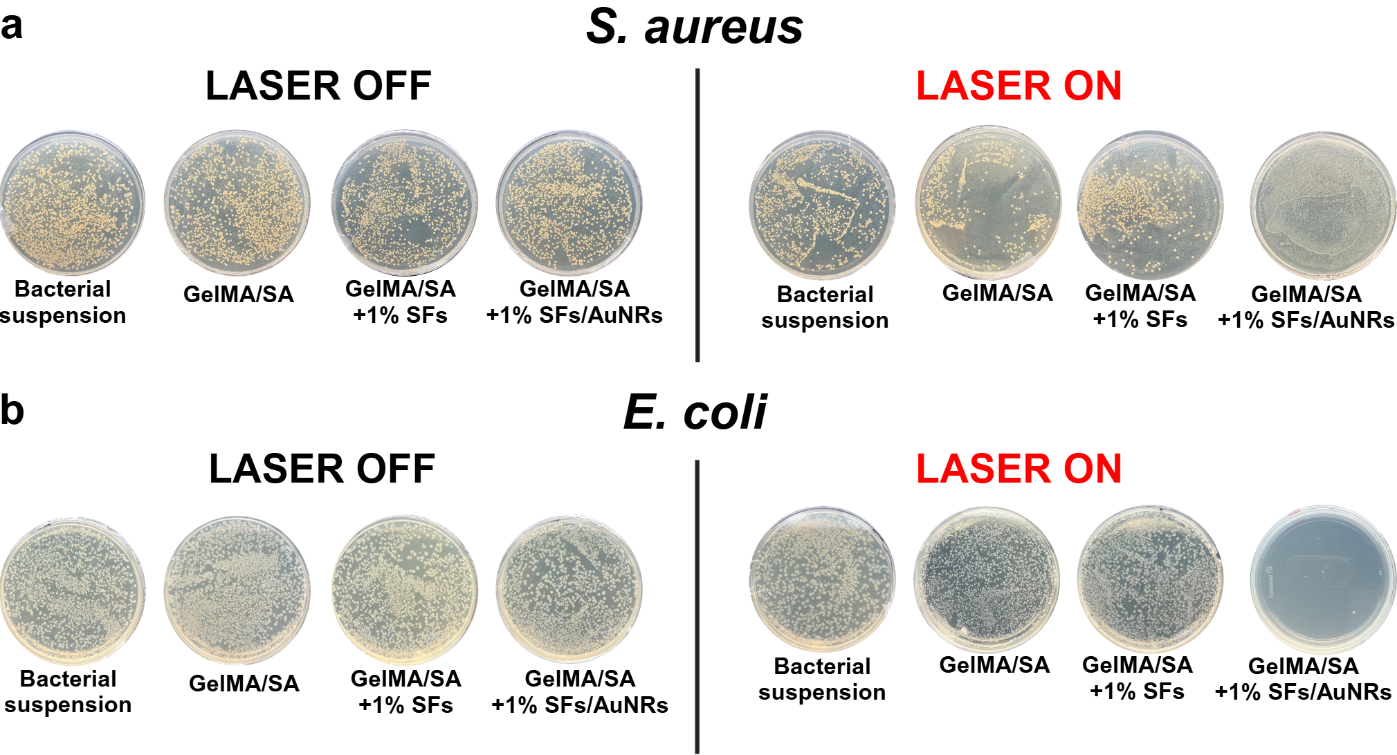


**Figure S7.** Photothermal inactivation of a) *S. aureus* and b) *E. coli* showing the representative photos of bacterial colonies grown on LB agar plates for each tested condition.

**
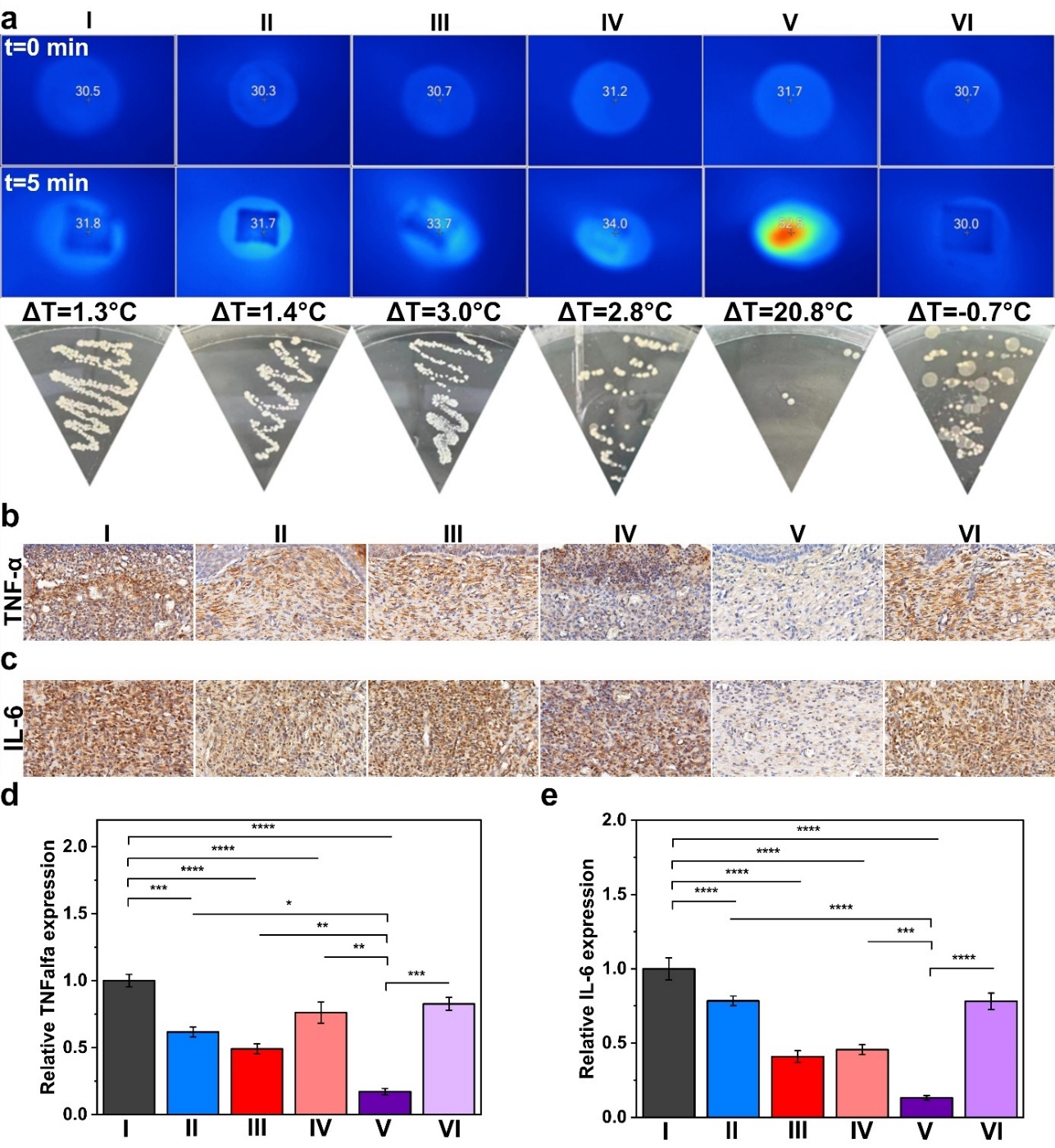
**

**Figure S8.** *In vivo* NIR treatment of infected wounds and immunohistochemical staining of wound tissues. a) Thermal images of samples before laser exposure and after 5 min of irradiation taken during *in vivo* anti-bacterial experiments, reached ΔT during test, and corresponding photos of bacterial colonies grown on LB agar plates for each tested condition. b) Immunohistochemical staining of pro-inflammatory cytokine TNF-alfa, c) IL-6, and corresponding to them, d) TNF-alfa positive area, and e) IL-6 positive area calculated using ImageJ. I – control group NIR **(+)**, II – GelMA/SA NIR **(+)**, III – GelMA/SA+1% SFs NIR **(+)**, IV – GelMA/SA+1% SFs/DXM NIR **(+)**, V – GelMA/SA+1% SFs/AuNRs/DXM NIR **(+)**, VI – GelMA/SA+1% SFs/AuNRs/DXM NIR **(-)**.
